# Evidence for symbolic use of ochre by Micoquian Neanderthals in Crimea

**DOI:** 10.1126/sciadv.adx4722

**Published:** 2025-10-29

**Authors:** Francesco d’Errico, Guilhem Mauran, Africa Pitarch Martí, Ana Majkić, Vadim Stepanchuk

**Affiliations:** ^1^ Université de Bordeaux, CNRS, MCC, PACEA, UMR 5199, Pessac 33615, France.; ^2^ University of Bergen, Centre for Early Sapiens Behaviour (SapienCE), Department of Archaeology, History, Cultural Studies and Religion, Bergen 5020, Norway.; ^3^ Rock Art Research Institute, University of the Witwatersrand, Johannesburg, South Africa.; ^4^ Departament d’Arts i Conservació-Restauració, Facultat de Belles Arts, Universitat de Barcelona, Barcelona, Spain.; ^5^ Institut d’Arqueologia de la Universitat de Barcelona (IAUB), Barcelona, Spain.; ^6^ Department of Archaeology, Faculty of Philosophy, University of Belgrade, Belgrade, Serbia.; ^7^ Institute of Archaeology of the National Academy of Sciences of Ukraine, Kiev, Ukraine.

## Abstract

Ochre use is widely regarded as a potential marker of symbolic behavior in Paleolithic societies. We conducted a multiproxy analysis of 16 ochre pieces from Middle Paleolithic Micoquian sites in Crimea [Zaskalnaya V (ZSKV), ZSKVI, and Prolom II] and mainland Ukraine (Mukhovets), spanning up to 70,000 years. Using portable x-ray fluorescence, scanning electron microscopy coupled to energy-dispersive spectroscopy, and technological analysis, we identified deliberate modifications including grinding, scoring, flaking, and scraping. Three pieces (ZSKV-05, ZSKV-06, and ZSKV-07) show features exceeding utilitarian use: One is shaped into a crayon-like tool with repeated resharpening, another appears to be a crayon fragment, and a third bears engraved, polished surfaces. These traits suggest the intentional production of marks and curated use. While practical applications (e.g., hide processing) remain plausible for other specimens, the evidence supports symbolic use among some Crimean Neanderthals. Our results highlight their cognitive complexity and underscore the importance of regional, multiproxy approaches in evaluating the emergence of symbolic material culture.

## Teaser

Technological and chemical analyses of Crimean ochres reveal modified pieces consistent with symbolic use by Neanderthals.

## INTRODUCTION

Over the past two decades, the utilization of ochre has become a crucial archaeological indicator for investigating the foundations of symbolic culture found across all contemporary and historically attested human societies (*1*–*5*). In all human cultures, the perception of reality is deeply shaped by the culturally embedded color systems (*6*–*8*). Colors influence language, rituals, body modifications, and many other practices that mold individual and collective identities (*9*–*11*). Nevertheless, the processes and mechanisms responsible for colors assuming such a central role in our existence remain inadequately documented and understood. A cultural colorization of reality may have occurred in human societies without leaving any archaeological trace. However, the deliberate use of coloring materials such as ochre, involving collecting them in the environment, processing them with appropriate techniques, and using them, possibly for different purposes, is generally considered an important clue to understanding when and how human societies made colors permanent and meaningful features of their cultures and, in particular, when colors became exosomatic tools for symbolic communication (*1*–*5*, *12*). Symbolism, in this context, refers to the human capacity to create and convey meaning through material proxies—such as colors or objects—that transcend their immediate practical functions. It reflects the ability to imbue these elements with shared, culturally specific significance, enabling communication, identity expression, and the transmission of abstract ideas across individuals and generations. Of particular interest are shaped ochre implements—especially crayon-like pieces bearing evidence of scraping, grinding, sharpening, and reuse—used to produce deliberate marks. These marks may have been applied to the skin, tools, or other surfaces and suggest that color was not merely applied but intentionally inscribed, transforming ochre into a medium for communication. This study contributes to understanding these developments by analyzing ochre pieces modified by Neanderthals in a region of Europe, Crimea, which has provided important but yet insufficiently studied evidences of ochre utilization. For the purposes of this study, we use ochre to refer to iron-rich mineral materials with red, orange, or yellow hues. When these materials show deliberate modification for powder production, we refer to them as coloring materials. The term pigment is reserved for cases where the use of ochre as a coloring agent—whether for utilitarian or symbolic purposes—can be inferred. We acknowledge that identifying ochre as a pigment or a marking tool implies a functional interpretation. To support these interpretations, we present converging lines of contextual, technological, and experimental evidence. The term symbolic use is applied only where this evidence substantiates the inference that ochre was used as a medium of meaningful expression. We explicitly lay out the evidence and reasoning for these interpretations in the following sections. This approach aligns with recent archaeological and ethnographic literature (*13*, *14*) while maintaining terminological precision. We proceed by first identifying ochre based on mineralogical and color characteristics. We then assess whether ochre was deliberately chosen, transported, and modified (e.g., ground, shaped, and engraved). Last, we interpret these modifications in the light of the material’s physical properties and contextual associations, particularly when they suggest repeated use, crayon-like shaping, or mark-making actions. While we do not assume that all ochre was used symbolically, where multiple lines of evidence converge, we argue that in those specific cases, symbolic use is the most parsimonious explanation.

**Fig. 1.**
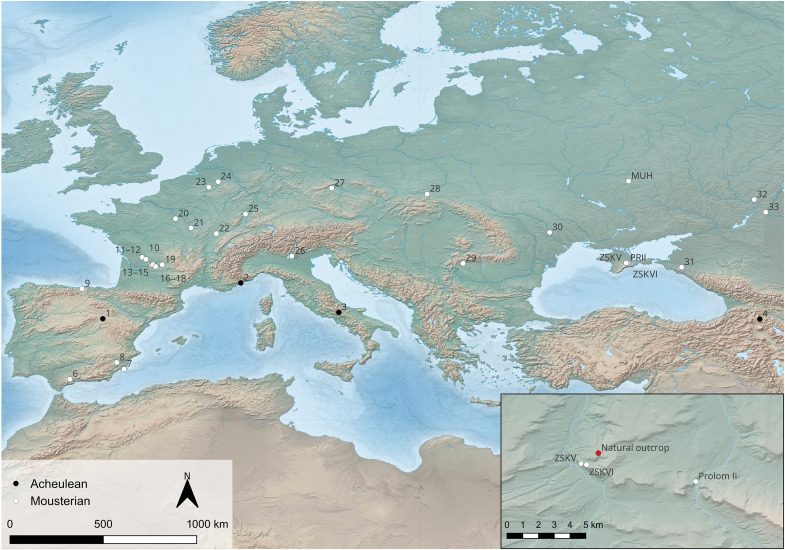
Ochre use at Lower and Middle Paleolithic sites and location of the studied sites in Crimea. Map showing Late Acheulean and Mousterian sites from Europe and western Asia with proposed or demonstrated evidence of ochre use. 1, Ambrona; 2, Terra Amata; 3, Isernia la Pineta; 4, Dashtadem 3; 5, Hungsi V; 6, Cueva de Ardales; 7, Cueva de los Aviones; 8, Cueva Antón; 9, Cueva del Castillo; 10, La Quina; 11, Tabaterie; 12, Station du Bonhomme; 13, La Ferrassie; 14, La Micoque; 15, Le Moustier; 16, Combe-Grenal; 17, Caminade-Est; 18, Pech-de-l’Azé I; 18, Pech-de-l’Azé IV; 19, La Chapelle-aux-Saints; 20, Ormesson; 21, Grotte du Renne; 21, Grotte du Bison; 22, Grotte de l’Ermitage; 23, Scladina; 24, Maastricht-Belvédère; 25, Achenheim; 26, Fumane Cave; 27, Beçov I; 28, Raj Cave; 29, Cioarei-Borosteni Cave; 30, Molodova 1; 31, Ilskaya 1; 32, Stalingradskaya; 33, Volgograd. Inset: Close-up of Crimea showing the location of the studied sites—ZSKV and ZSKV and PRMII—along with the natural outcrop, called Red Gully, sampled for this study. The Red Gully source, located ~1.5 km from the ZSK sites, is analyzed here to explore its potential use by Neanderthals.

Contrary to what was still believed a quarter of century ago, archaeological evidence suggests that rather than corresponding to a sudden changeover, the use of ochre by human cultures was the result of a slow evolution, with ancient roots, involving different fossil human species. The earliest materials that could have been used as pigments, consisting of different types of rocks rich in iron oxides, commonly known as ochre, date back at least 400 thousand years (ka) in Africa, as in Europe. However, because of its attested or supposed use in utilitarian activities (*15*–*17*), ochre is seen by many authors as an ambiguous element of the archaeological record, which cannot be interpreted by, and in itself, as proof that this material was used symbolically, without additional compelling evidence, when it is first recorded in the archaeological record. Other authors (*18*, *19*) point out that symbolic and utilitarian functions are intimately linked among traditional populations and that, as a result, it would have been difficult for a systematic use of ochre powders to exist over a long period of time without a symbolic dimension being rather quickly attached to it. To find a way of choosing between these contrasting interpretations, several studies have set out to characterize the behaviors associated with the exploitation of mineral colorants. These studies were based on the idea that a better understanding of these processes can document the degree of behavioral complexity associated with their use and support or contradict their utilization for symbolic purposes. The gradual increase in the use of coloring minerals in the archaeological record (*5*), the preference for certain colors (*20*), the search for particular raw materials from distant sources (*21*–*23*), the heating of certain rocks to change their color (*19*, *21*, *24*, *25*), the application of coloring compounds to change the color of objects used as personal ornaments (*26*–*29*), the production of abstract engravings on ochre pieces (*30*, *31*) or of abstract drawing with ochre crayons (*32*), and the production of small quantities of coloring powder (*33*) considered as better suited for symbolic rather than utilitarian activities are the evidence most frequently cited to support the hypothesis that coloring powders were involved in symbolic practices very early in time. The recent synthesis by Dapschauskas and colleagues (*5*) identifies 87 African sites, concentrated in Northwest and eastern South Africa, dated between 500 and 40 ka, which have yielded more than 25,000 pieces of ochre. Information about these ochre fragments are, however, variable and, in many cases, compelling evidence that ochre was deliberately brought to the site and used is lacking. By examining the chronology of their occurrence, the authors identify three phases of ochre use in the African Middle Stone Age, an “initial” phase from 500 to 330 ka, an “emergent” phase from 330 to 160 ka, and a “habitual” phase from 160 to 40 ka. The authors interpret the latter phase, when a third of archaeological sites contains ochre, as the material manifestation of intensifying ritual activities in early populations of *Homo sapiens*. This view is consistent with the systematic discovery of ochre residues on marine shells used as personal ornaments at sites from Northern and Southern Africa dated to 140 to 70 ka (*28*). The presence of residues on the whole shell surface is interpreted as evidence that, at least in some instances, the shells were deliberately coated with red ochre while being worn, a practice supporting a symbolic use of this material (*26*). In Eurasia and particularly in Europe, numerous pre–Upper Paleolithic sites are reported in the literature as having yielded ochre (Fig. 1 and table S1).

However, as in Africa, many of these findings lack crucial information to confirm the anthropogenic origin of the ochre and their specific use at the sites. The earliest evidence for the use of ochre in Europe comes from the Acheulean site of Terra Amata, Nice, France, where 77 pieces of ochre were found in archaeological layers dated to ~380 ka. Several of these pieces bear ground facets or traces of scraping, and it has been proposed that some may have been heated to change their color (*34*). Less convincing is the reported use of ochre at the Acheulean site of Ambrona, Spanish Meseta, dated to around 350 ka (*35*). Hematite-soaked sediment lumps have been identified in archaeological levels dated to 250 ka at Maastricht-Belvedere, the Netherlands (*3*). A modified piece of ochre was found at the contemporary site of Achenheim, France (*36*). Beçov I, in the Czech Republic, dated to the same period (250 to 200 ka), is thought to have yielded friable rocks of several colors and tools to ground them (*37*–*39*). Numerous Mousterian sites from Europe have yielded rock fragments rich in manganese oxides, modified by grinding and scraping, as well as pieces of ochre modified using the same techniques (Fig. 1 and table S1). The traces of modification recorded on these materials do not differ substantially from those observed at contemporary and older African sites.

The best-known Mousterian sites from Europe that have yielded evidence of ochre use include Combe-Grenal (*40*), Ormesson (*41*), Pech de l’Azé (*42*–*44*), Le Moustier (*42*, *45*), La Micoque, La Chapelle–aux-Saints, La Grotte de l’Ermitage, la Quina, and la Ferrassie (*46*) in France; Cueva de los Aviones, Cueva Antón (*47*), Cueva del Castillo, and Cueva Morín (*48*) in Spain; and the Fumane cave in Italy (*49*). The last Neanderthals of Western Europe, to whom archaeologists attribute the making between 45 and 42 ka of the so-called Châtelperronian technocomplex, used large quantities of red ochre and black manganese oxides (*50*, *51*). The identification of ochre residues on numerous bone tools found in the Châtelperronian layers of the Grotte du Renne, Arcy-sur-Cure, including bone awls and lissoirs interpreted respectively as tools to pierce and soften hides, suggests that an ochred compound was used for tanning and possibly coloring the clothing of the last Neanderthals (*50*, *52*). Manganese oxides found in the same layers have been variously interpreted as substances used for body painting (*53*) or as fire accelerants (*54*), with traces of use and experimental evidence supporting both hypotheses. A symbolic use of ochre by Neanderthals has been inferred from its application to produce pigments used to color marine shells at Cueva Antón (*47*) and Fumane (*49*), as well as to decorate a stalagmitic dome with red markings at Cueva de Ardales (*55*).

The possible earliest evidence of ochre use in Asia comes from Dashatadem-3, Armenia, where four pieces of ochres were found in a Late Acheulean context (*56*). Fragments of “hematite,” interpreted as having been brought to the site from geological sources located 25 km from it were found at the Acheulean site of Hungsi locality V, North Karnataka, India (*57*).

At Jwalapuram locality 3 (Andhra Pradesh, southern India), a fragment of red ochre bearing traces of grinding was found in an archaeological layer underlying the Toba ashes and dated to ~77 to 74 ka (*58*). In Dhaba, Locality 1, ochre fragments were found in a 78- to 65-ka-old layer (*59*). With the only exception of the Madjedbebe site in Northern Australia, which has yielded red and yellow ochre pieces in layers dated to between 65 and 52 ka, the 21 other South-East Asian sites in which ochre fragments were found are more recent than 50 ka (*60*).

To date, very few Russian sites attributed to the Middle Paleolithic have provided compelling evidence of ochre use (*61*). Spots or clusters of ochre and ochre residues on artifacts are reported in archaeological layers at several Early Upper Paleolithic sites in Siberia, dated from 43 to 35 ka (*62*, *63*). The oldest piece of Siberian ochre with traces of grinding is believed to have been found in the Early Upper Paleolithic layers of Denisova, possibly dated at 50 ka, but not yet formally published (*64*). The earliest possible evidence for ochre use in China consists of microscopic ochre residues identified inside engraved lines on a bone found at Lingjing, Henan, a site dated to ~125 ka, possibly inhabited by Denisovans (*65*). Modified and unmodified ochre fragments, tools for processing ochre, and objects bearing ochre residues are reported from 15 Chinese Early Upper Paleolithic sites ranging from 40 to 12 ka (*66*, *67*).

### The Crimean evidence

**Fig. 2.**
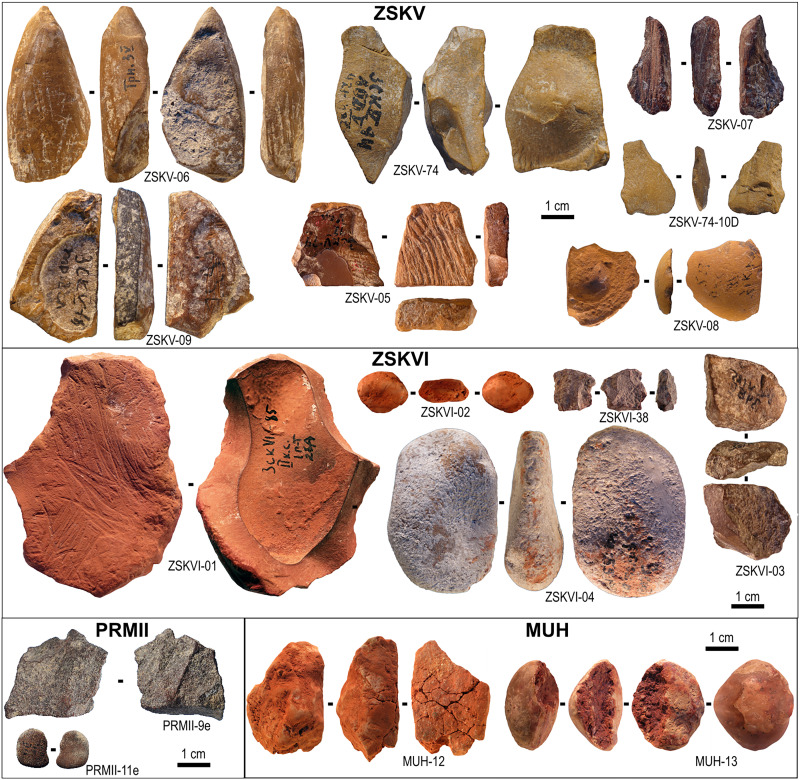
Archaeological ochre pieces from Crimea. Studied archaeological coloring materials from ZSKV, ZSKVI, PRMII and MUH.

In this study, we focus on ochre use by Neanderthals associated with the Crimean Micoquian—a regional variant of the Mousterian technocomplex characterized by bifacial tools, particularly bifacial backed knives. The Mousterian, more broadly, refers to the Middle Paleolithic cultural and technological tradition typically associated with Neanderthals across Europe and parts of western Asia. Although largely ignored in the Western literature on Neanderthal ochre use, the utilization of this material by Micoquian Crimean Neanderthals has long been reported. Already in 1983, Kolosov published an ochre fragment from Zaskalnaya VI (ZSKVI), layer II, bearing traces of scraping (*68*, *69*). An ochre pencil from ZSKV trench and a piece of ochre with signs of scraping and engraving from ZSKV, layer V, were subsequently identified in the collections from previous excavations by one of us (V.S.) in the early 2000s (*70*). The same author also mentions the presence of 44 ochre pieces in the Micoquian layers of Prolom II (PRMII), most of which interpreted as bearing traces of modification and use (*71*). The analysis of red residues on stone tools from the Late Acheulean context at ZSKIX has led the authors to conclude that ochre was used at this site for utilitarian purposes (*72*). In a more recent paper (*73*), Stepanchuk describes eight pebbles bearing red residues from ZSKV trench; ZSKVI, layers I, II, III, II to IV, and V; and PRMII, layer III, and interprets one of them as presenting an accidental red stain and the others as ochre processing tools or objects intentionally covered with ochre. In the same paper, the author mentions the presence of 178 pieces of ochre bearing traces of human activity and ochre-stained lithic, bone, and human remains from the Micoquian layers of ZSKV, ZSKVI, and PRMII. Ryzhov and colleagues (*72*) report ochre use in hafting adhesives at the Late Acheulean site of ZSKIX. Currently, the total number of ochre and ochre-stained items reported in Crimean Middle Paleolithic assemblages is 291 pieces, although this figure includes numerous fragments whose identification as deliberately transported and anthropogenically modified ochre has not been systematically confirmed. This brief description nevertheless highlights the exceptional potential of the Crimean record to document and better understand Neanderthal cultural practices involving the use of ochre. The interest of this record is further supported by the fact that we are dealing with a constellation of Paleolithic sites that are geographically proximate to each other. Within a radius of 2 km, one finds the rock shelters of Ak-Kaya I to V, ZSKI to ZSKIX, and Karabai II, alongside the open-air sites of Krasnaya Balka, Karabai I, and Sary Kaya I to IV. This clustering of sites presents an intriguing opportunity to explore how ochre sources may have changed over time and to consider potential connections to distinct Micoquian facies or subfacies—a perspective that invites further research and opens avenues for future investigation. Here, we present a technological, compositional, and functional analysis of unpublished ochre pieces (see Materials and Methods) from key Crimean and one non Crimean sites and compare their chemical composition with a local ochre source in Crimea. Sixteen archaeological ochre pieces were analyzed in the present study. Seven pieces come from ZSKV, five from ZSKVI, two from PRMII, and two from Mukhovets (MUH) (Table 1, Fig. 2, and table S2). These two last objects were included for comparative purposes, as the site is associated with a Micoquian technocomplex and provides an opportunity to explore regional differences in ochre selection and processing. Both pieces were selected on the basis of their well-preserved surfaces and potential evidence of anthropogenic modification. The analyzed samples also include four natural ochre fragments from an Oligocene clay formation within the Maykop Series, specifically from clay strata exposed at the Plateau above Red Gully (45°07′N, 34°37′E), an outcrop located 1.5 km northeast of the ZSK sites. The results presented here are particularly valuable given the current political situation in Crimea, which complicates access to these important archaeological sites and the ability to conduct new fieldwork in the region.

**Table 1.** Data on archaeological ochre pieces and description of the modification traces visible on each sample. R, red; DR, dark red; B, brown; Y, yellow; Morph, morphology; S, slab; P, pebble; C, chunk; Cr, crayon; I, irregular; F, flake; Scr, scraping; Sco, scoring; Gr, grinding; Sm, smoothing; Gr. ind., grinding index; Fla, flaking; Per, percussion marks; Fr, fracture; Fr. ind., fracture index; Postdep, postdepositional; a, transport smoothing; b, probable engraving; Mod. ind., modification index.

Site	Sample ID	Layer	Color	Lithology group	Length (mm)	Width (mm)	Thickness (mm)	Morph	Scr	Sco	Gr	Sm	Gr. ind.	Fla	Per	Fr	Fr. ind.	Mod. ind.	Postdep
ZSK-VI	ZSKVI-01	I/II	R	1a	62.6	47	19.8	S	0	1	0	1(a)	2a	1	1	0	2	4a	0
	ZSKVI-04	II	R	2	41.9	27.9	14.9	P	0	0	0	0	0	0	0	0	0	0	1
	ZSKVI-03	II	DR	3	20.8	16.7	6.3	C	0	0	0	0	0	1	0	0	1	1	0
	ZSKVI-02	II/IIIa	R	1a	14	11.8	6.8	P	0	0	?	0	0	0	0	0	0	0	0
	ZSKVI-38	IIIa	B	4	13	12.6	6.4	C	0	0	0	0	0	1	0	0	1	1	0
ZSK-V	ZSKV-06	Trench	Y	1b	44.8	23.3	11.8	Cr	1	0	1	1	3	1	1	1	3	6	0
	ZSKV-09	II	Y	1b	33.2	20.4	8.9	S	0	0	0	0	0	0	0	0	0	0	1
	ZSKV-05	V	R	1a	23.9	24.1	6.2	S	0	1(b)	1	1	3b	1	0	1	2	5b	?
	ZSKV-07	V	DR	4	25.4	11.8	7.5	Cr	1	0	1	0	2	0	0	1	1	3	0
	ZSKV-74	V	Y	1b	40	26.5	18.7	I	0	0	0	0	0	1	0	0	1	1	1
	ZSKV-10D	V	Y	1b	23.5	16.2	5.6	I	1	0	0	1(a)	2a	1	0	0	1	3a	1
	ZSKV-08	VI	Y	1b	26.2	24.1	5.4	F	0	1(b)	1	0	2b	1	1	0	2	4b	0
PRMII	PRMII-9e		B	4	24	23.1	7	C	0	0	0	0	0	1	0	0	1	1	0
	PRMII-11e		B	4	12.6	10	3.4	P	0	0	1	0	1	0	0	0	0	1	1
MUH	MUH-12	680–700	R	5	31.3	25.9	14	P	0	0	0	0	2	?	0	0	0	0	0
	MUH-13	700–720	R	6	30.1	27.2	19	I	1	1	0	0	0	0	1	0	0	3	0

The approach applied to the study of this material enables to identify where ochre was collected by Micoquian Neanderthal in Crimea and how it was modified and used. Our results support the hypothesis that ochre was actively involved in symbolic practices.

### Archaeological context

We focus on three Micoquian Middle Paleolithic sites in Crimea—ZSKV, ZSKVI (Kolosovskaya), and PRMII—dated between marine isotope stage (MIS) 5c (~100 to 90 ka) and ~33 to 36 thousand calibrated years before the present (ka cal B.P.). All three sites are attributed to the Ak-Kaya facies of the Micoquian technocomplex, characterized by bifacial tools including backed knives and leaf points. Here, we analyze a curated sample of 14 ochre pieces from these sites, selected on the basis of preservation and evidence of modification. Two additional pieces from MUH, a Mousterian site in northern Ukraine, are included for regional comparison. ZSKV, ZSKVI, and PRMII are rock shelters located in the Belogorsk region. Both have yielded rich lithic and faunal assemblages, Neanderthal remains, and multiple stratified layers containing ochre. The analyzed ochres from ZSKV come from layers II, V, and VI and the trench unit; those from ZSKVI derive from layers I/II, II, and IIIa. PRMII, situated ~6.5 km away, has also produced numerous mineral colorants, particularly in its upper cultural layers. MUH provides additional comparative material from outside Crimea. Details on sites’ stratigraphy, excavation history, and broader archaeological context are presented in text S1.

### Sedimentological context and implications for ochre provenance

All ochre artifacts analyzed in this study were recovered from the typical cave sedimentary infill characterizing the three Crimean sites under consideration. This infill is composed predominantly of pale loam with a variable admixture of poorly rounded gravel (1 to 5 mm in size), derived from the in situ weathering and erosion of nummulitic limestone bedrock. The sediment matrix contains up to 15% silt and includes a minor component of nummulitic limestone sand. Geochemical data and sedimentological observations indicate that iron-bearing minerals, such as magnetite ( ${\text{Fe}}^{2+}{\text{Fe}}_{2}^{3+}O_{4}$ ), occur only in small amounts (~4% of total composition), consistent with the dispersed iron content typical of nummulitic limestones. The sediments lack macroscopic fragments of naturally occurring ochre. The only natural iron oxide admixtures present are in the form of microresidues or diffuse punctate pigmentation, which do not account for the presence of the larger ochre pieces documented in the archaeological layers. These observations support the conclusion that the ochre fragments described in this study were not introduced by natural processes but rather through deliberate collection and deposition by Neanderthal occupants.

## RESULTS

### Technological and functional analysis

#### Zaskalnaya VI-01

**Fig. 3.**
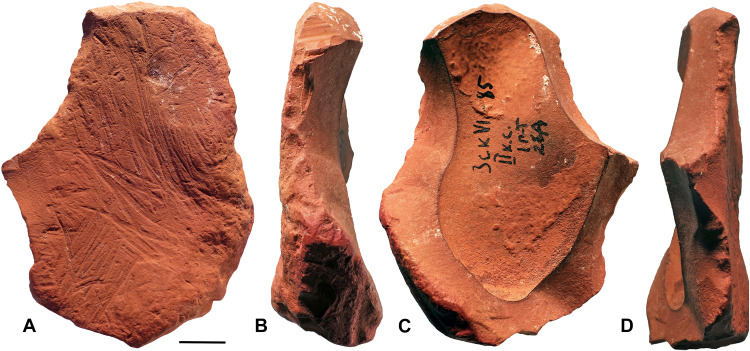
Modified ochre fragment (ZSKVI-01). Four views of ochre fragment ZSKVI-01 displaying multiple modifications. (**A**) Outer friable surface exhibiting extensive scoring, pitting, and short randomly oriented striations on smoothed areas. (**B**) Right margin showing heavily worn microchipping. (**C**) Concave, harder, inner surface displaying on its right margin. (**D**) Fresh invasive flake scars. Scale bar, 1 cm.

The fragment is an iron-rich crust with a flat outer surface covered by a millimeter-thick layer of friable ochre and a concave harder inner surface partially coated with a thin, irregular red concretion (Fig. 3C). The material is predominantly homogeneously orange, with a prominent dark-red layer along part of its lower left edge. The outer surface (Fig. 3A) bears extensive evidence of scoring, with three main groups of striations oriented in distinct directions. These striations vary in form and depth—straight, curved, and S-shaped lines—and suggest deliberate, robust actions carried out by different points with the clear intent of removing red coloring powder. Beyond the primary scored areas, the remainder of the surface features more superficial, shorter, and randomly oriented striations, which appear smoothed, likely from previous wear due to transport. Both areas feature small pits due to impacts. The edges of the fragment display distinct phases of modification (Fig. 3, B and D). On the right edge of the inner surface, the flake removals are invasive and fresh, indicating relatively recent actions. They are coded as postdepositional modifications in Table 1. In contrast, the left edge of the outer surface exhibits marginal flake removals and microchipping that appear heavily smoothed, likely because of extended use or transport. In addition, small patches of white concretions are visible on both surfaces, reflecting postdepositional processes or environmental exposure over time. The overall evidence recorded on ZSKVI-01 suggests a palimpsest of actions. The fragment initially underwent a first generation of contiguous, superficial flake removals on one edge, which were subsequently heavily smoothed by use or transport, a process that also affected the friable outer surface. This was followed by deliberate scoring of the friable surface to extract fine red powder and, later, the removal of larger invasive flake scars on the opposite edge. These multiple phases of modification suggest a well-organized strategy involving the application of scoring actions to friable ochre for the production of fine coloring powder and knapping (coded as flaking in Table 1) of harder ochre to generate small flakes, which were likely pounded to produce coarser ochre powder. The pits recorded on the outer surface may be due to contact during transport, with harder objects, such as lithics, before disposal of the object.

#### Zaskalnaya VI-04

ZSKVI-04 is a pebble almost completely coated with a whitish, granular calcium carbonate concretion (Fig. 2). In areas where the coating is absent, a light brown alteration surface is visible. Where this alteration layer is missing, it reveals a bright-orange sandstone. Superficial traces of grinding, affecting both the concretion and the inner layers, may be postdepositional in origin. Thus, we considered that no anthropogenic modifications could be seen on this ochre piece (Table 1).

#### Zaskalnaya VI-03

ZSKVI-03 is a piece of hard rock, brown in color and noticeably heavier than the other ochre pieces found at the site (Fig. 2). The fragment appears to result from knapping and smashing activities (percussion marks and flaking in Table 1), likely to recover flakes for crushing to produce a darker ochre powder.

#### Zaskalnaya VI-02

ZSKVI-02 is a piece made of soft, powdery light orange ochre featuring a thin layer of quartz in its middle (Fig. 2). The faint traces of possible facets due to intentional grinding are visible on the edge of the piece, but they lack diagnostic oriented striations typical of this technique due to the friable nature of the raw material.

#### Zaskalnaya VI-38

ZSKVI-38 is a flat chunk of iron-rich rock displaying multiple removals on both aspects, suggesting that it was purposefully knapped to produce chips subsequently pounded to produce ochre powder (Fig. 2). A small postdepositional fracture exposing the inner material reveals a bright-red ochre, strengthening its identification as a desirable ochre source.

#### Zaskalnaya V-06

**Fig. 4.**
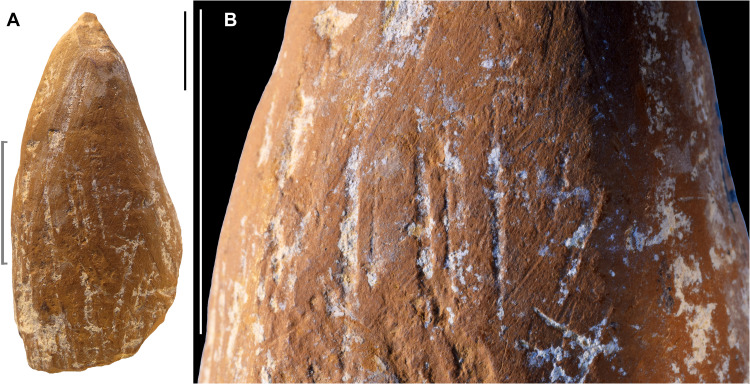
Scraped and ground ochre crayon (ZSKV-06). Ochre fragment ZSKV-06. (**A**) General view. (**B**) Macrophoto on the central portion showing rough scraping marks aligned with the object’s main axis, partially removed by oblique striations from grinding. Scale bars, 1 cm.

ZSKV-06 is an elongated fragment of yellow ochre, fully shaped into a crayon-like tool with a pointed morphology (Fig. 2). The artifact exhibits a palimpsest of traces reflecting a sequence of shaping and utilization. Initially, the piece was roughly shaped by scraping to create its general form (Fig. 4).

**Fig. 5.**
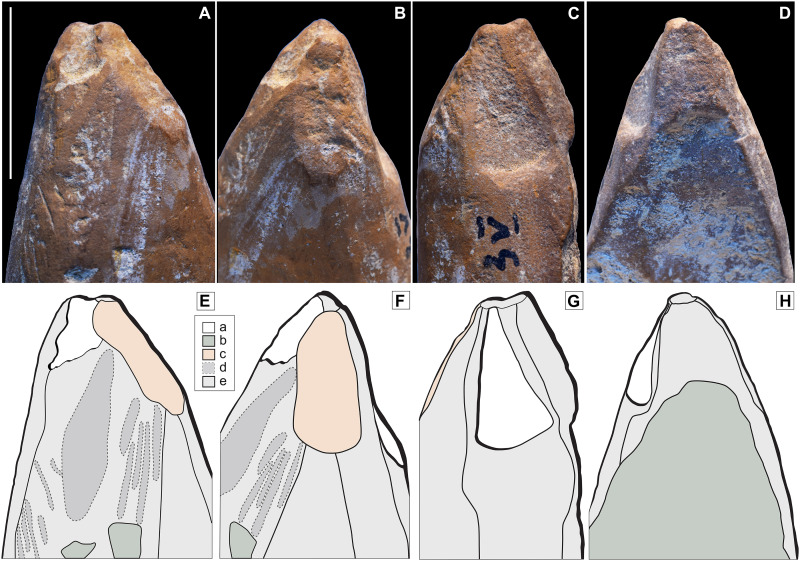
Tip of an ochre “crayon.” Macrophotographs of the four sides of the ochre fragment ZSKV-06 tip (**A** to **D**) and corresponding drawings (**E** to **H**), illustrating patterns distinguishing anthropogenic and accidental modifications. Key: (a) Burin-like flake scars with the edges of flake scar in G partially removed by subsequent grinding; (b) breakage; (c) concave facet produced by gouging; (d) scraping marks smoothed by subsequent grinding; (e) adjacent facets produced by grinding. Scale bar, 1 cm. Drawings: L. Geis, PACEA, University of Bordeaux.

This was followed by finer grinding on a grindstone, which largely erased the scraping marks, except in a few areas far from the tip. Subsequent modifications indicate that the tool was periodically rejuvenated (Fig. 5). Burin-like removals originating from the tip suggest use or intentional sharpening to maintain its point. These removals were carefully reworked through further grinding and gouging, ensuring that the tip remained functional. While some removals were intentional to enhance sharpness, others may reflect wear from contact with a surface under applied pressure. The current state of the tip, which is partially broken and has a blunt edge, suggests that it was used after the last sharpening by grinding, likely on a soft material, given the ochre’s relatively low hardness.

At a later stage, the tool sustained a longitudinal fracture that removed a substantial portion of one aspect while preserving the tip. This tool appears to have functioned as a marking tool, akin a pencil, used to produce lines on a surface. The combination of shaping, maintenance, and wear traces underscores its curated nature and sustained utility as an instrument for drawing or marking.

#### Zaskalnaya V-09

ZSKV-09 is a fragment of flat, iron-rich material consisting of a convex outer layer of red ochre and a concave inner layer of yellow ochre (Fig. 2). The red surface features deeply incised, longitudinally oriented, subparallel lines, which appear to have been made recently and thus coded as postdepositional (Table 1).

#### Zaskalnaya V-05

**Fig. 6.**
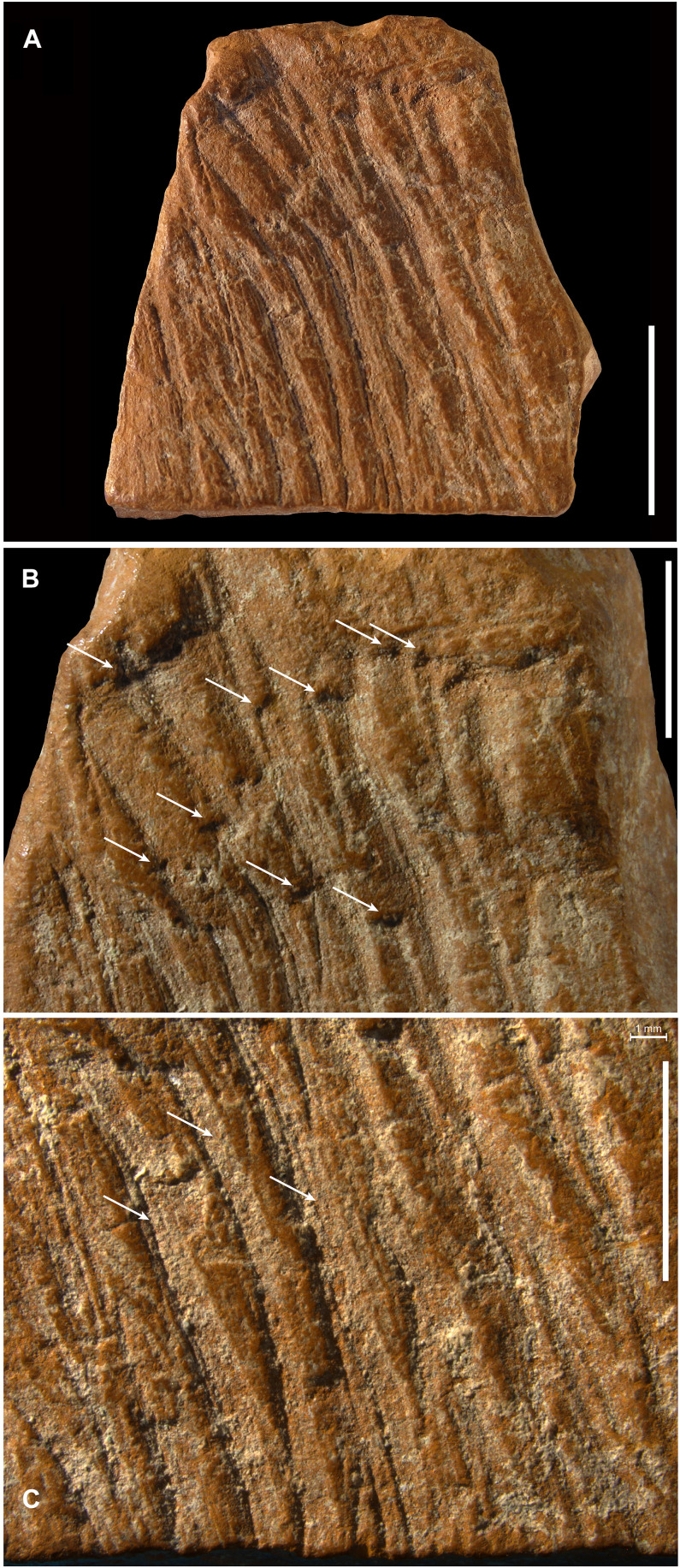
Ochre fragment ZSKV-05. (**A**) Flat surface modified with deep, slightly curved, subparallel incisions. (**B**) Close-up of the narrow edge showing pits (arrows) at the starting points of the incised lines, formed by the pressure of the incising tool. (**C**) Microscopic view revealing diagnostic parallel, equally spaced grooves (arrows) indicating the use of the same tool, along with smoothing of the prominent areas between the incisions, suggesting postmodification handling and curation. Scale bars, 1 cm (A) and 5 mm [(B) and (C)].

ZSKV-05 is a flat fragment of friable ochre, orange in color, with one flat surface covered by a thin, darker, and harder layer (Fig. 2). The opposite surface has been entirely modified through the incision of deep, slightly curved, subparallel lines (Fig. 6A). Microscopic analysis reveals that the lines were all created by moving the tool consistently in the same direction, starting from the narrower edge of the slab and progressing toward the wider edge. The starting points of the lines, marked by pits formed from the pressure of the incising tool, are clearly visible near the narrow edge (Fig. 6B). The analysis also indicates that the lines were made using the same tool, which left diagnostic traces in the form of parallel, equally spaced grooves (Fig. 6C).

The cross sections of the lines suggest the incisions were made by a right-handed individual, as evidenced by the abrupt wall on the left side of the lines. Right handedness is further supported by a shift in the direction of the lines when they cross a natural discontinuity in the surface, where the lines skip to the right. The pattern suggests an apparent intention to create an organized design, reflected in the deliberate effort to avoid overlapping the lines and to maintain their parallel arrangement. The prominent areas between the incised lines are highly smoothed, suggesting that the object was handled and curated after being modified. Smoothing was thus coded in Table 1 as the result of transport. One edge of the slab was carefully ground to create an elongated, flat surface covered with fine oblique striations. A small area of this ground facet was later removed through flaking. Removals due to flaking are also identified on the face opposite to the incised surface.

#### Zaskalnaya V-07

**Fig. 7.**
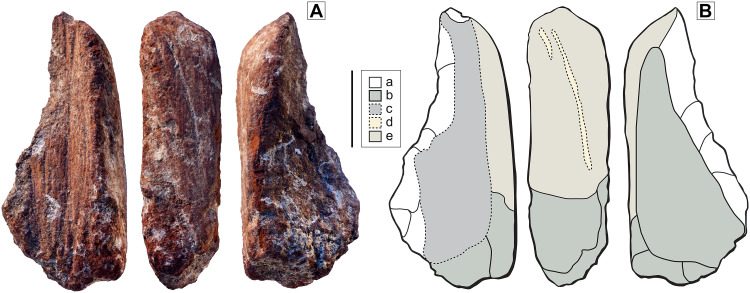
Ochre fragment ZSKV-07. (**A**) Photographs of three aspects of the fragment. (**B**) Drawing identifying key modifications. Key: (a) Flake scars; (b) natural breaks; (c) intense to-and-from incising, producing a deep V-shaped longitudinal groove; (d) residues of scraping marks removed by grinding; (e) convex ground surface, interpreted as the remnant of the original ochre crayon surface. Scale bar, 1 cm. Drawings: L. Geis, PACEA, University of Bordeaux.

This specimen is interpreted as a fragment of a larger, deliberately shaped red ochre piece that originally had a crayon-like morphology (Fig. 2). Technological analysis suggests that the initial shaping involved, as in specimen ZSKV-06, scraping followed by grinding, producing convergent facets along a convex edge—traces of which are still visible despite heavy surface smoothing (Fig. 7). The preserved portion likely represents the basal segment of a pointed crayon, the tip of which is now missing. After breakage, the longitudinal fracture surface was modified by the incision of a deep, V-shaped groove, likely intended for the extraction of coloring powder. This groove was subsequently altered through flaking, which removed part of its profile and created a large notch on the fragment. It is possible that this flaking aimed to produce small ochre flakes, which were then pounded to obtain red powder.

#### Zaskalnaya V-74

ZSKV-74 is a chunk of smoothed yellow siltstone, likely rich in goethite, naturally detached from a larger block through weathering, probably due to frost shattering, as suggested by a characteristic conchoidal fracture visible in the middle of one aspect (Fig. 2). The remainder of the surface displays several fresher flake removals, produced with the intent of creating small flakes to be pounded into yellow powder and thus coded as flaking in Table 1.

#### Zaskalnaya V-74_10D

ZSKV-74_10D is a small flake of yellow siltstone, likely rich in goethite, displaying numerous dorsal flake scars indicative of earlier removals before the flake’s detachment, produced with the likely intent to generate flakes to be pound to produce yellow coloring material (Fig. 2). The distal portion of the dorsal aspect exhibits heavily smoothed traces consistent with longitudinal scraping. The ventral aspect shows marginal accidental breaks, likely due to the thinness of the flake edges, along with an association of micropits and short, randomly oriented striations.

#### Zaskalnaya V-08

**Fig. 8.**
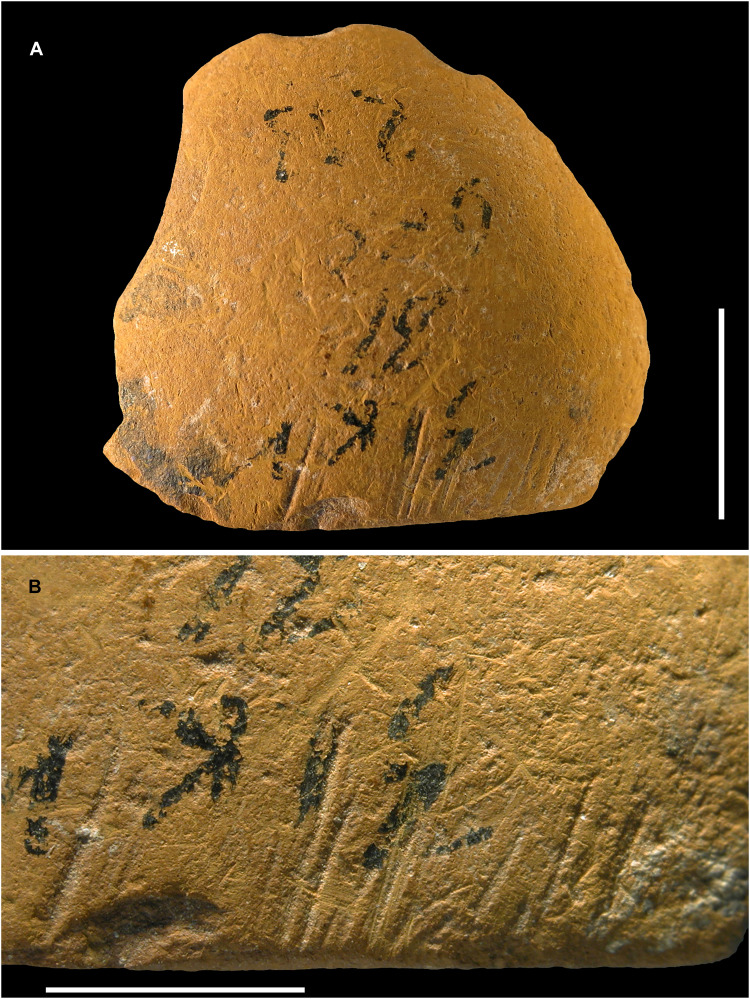
Ochre fragment ZSKV-08. (**A**) Photograph of the fragment, showing its outer surface with evidence of impact marks. (**B**) Close-up of the edge with a series of intentionally made subparallel incisions. Scale bars, 1 cm (A) and 5 mm (B).

ZSKV-08 is a fragment from the outer layer of a pebble composed of dark-yellow siltstone, likely rich in goethite (Fig. 2). The inner surface exhibits signs of chemical etching, visible as micropits, while prominent areas appear slightly flattened from grinding. The outer surface is marked by a palimpsest of randomly oriented short striations and impact pits. Near one edge, a series of about a dozen subparallel incisions was intentionally made (Fig. 8). These were coded as scoring in Table 1. A deep incision located close to an end is recent in origin and thus considered as postdepositional (Table 1).

#### Prolom II-9e

PRMII-9e is a small flake, dark-brown in color (Fig. 2). A small damaged area exposes the inner material, which reveals a bright-red color and a fine-grained texture, making it suitable for the production of red mineral pigment. The dorsal aspect bears multiple flake scars, indicative of earlier removals before the flake’s detachment, suggesting their use, after pounding, to produce red ochre and coded as flaking in Table 1.

#### Prolom II-11e

PRMII-11e is a small flat pebble with a dark-red patina (Fig. 2). Areas with recent scratches reveal a red/orange inner ochre. One face and one edge display parallel striations produced by grinding (Table 1).

#### Mukhovets-12

MUH-12 is a highly heterogeneous, rounded fragment of powdery reddish ferruginous sandstone containing large calcareous clasts and microfossils (Fig. 2). Any traces of modification that may have been originally present have not survived the degradative processes acting on the material. It is interpreted as bearing no modifications.

#### Mukhovets-13

MUH-13 is a subcircular object with a glossy appearance, featuring one flat and one convex surface (Fig. 2). The convex side retains a small appendix near the edge, a V-shaped groove at the center, and exhibits bilateral symmetry, suggesting that it is a fossil—likely the internal cast of a crab’s cephalothorax. The appendix features a rostrum, while the V-shaped groove represents the internal impression of the cervical groove. The convex surface shows signs of superficial scraping. Approximately half of the flat surface has been removed, possibly by knapping and scoring, revealing the fossil’s infilling. This infilling consists of large quartz grains bound together by a dark, bright-red, iron-rich matrix.

### Macroscopic and microscopic characterization of ochre materials

The analyzed samples exhibit a diverse range of compositions and morphologies, encompassing red and yellow claystone, iron crusts, ferruginous carbonates, and various forms such as slabs, pebbles, chunks, flakes, shaped crayons, or irregular fragments (Table 1). Their dimensions range from 10 to 62 mm (Fig. 2 and Table 1). Notably, sample ZSKVI-04 is distinguished by a notable postdepositional calcitic crust that can obscure original use traces. Optical and scanning electron microscopy (SEM) (figs. S1 and S2 and table S4) reveal the following textures and subtextures: (i) fine-grained ferruginous claystone composed of small globular to irregular iron oxide/hydroxide particles dispersed in a clay matrix together with irregular angular quartz (fig. S2, A and E to I): (ia) Enriched with hematite, resulting in a red hue, and (ib) enriched with goethite, contributing to a yellow hue, with minor calcite infiltration; (ii) postdepositional crusted claystone, most probably similar to ia but extensively covered by a postdepositional calcitic crust made of an aggregate of needle-like calcite (fig. S2B); (iii) dark-red ferruginous claystone, fine-grained texture mainly composed of irregular aggregated iron oxide particles with notable quartz inclusions (fig. S2C); (iv) granular hematite containing quartz inclusions, with hematite acting as a cementing agent; (v) ferruginous limestone, characterized by poorly sorted fossil inclusions; (vi) fossilized ferruginous limestone, with possible fossil structures.

Optical and electronic microscopy confirmed the macroscopic descriptions, revealing dispersed iron particles within clay matrices in most samples (table S4). Quartz inclusions were observed in several specimens, such as ZSKVI-02, while calcitic crusts of varying thicknesses cover many samples. Distinct outliers include iron crust (ZSKVI-07), which exhibits a massive texture; hematite fragments (PRMII-9e and PRMII-11e), composed of a hematite matrix with randomly oriented quartz inclusions; ferruginous carbonates (MUH), a sample (MUH-12) features unidentified fossil structures, while another (MUH-13) appears to correspond to a ferruginous fossil.

### Elemental composition

The archaeological samples and those coming from the natural outcrop exhibit significant variations in their chemical composition, consistent with macroscopic and microscopic observations (table S6). The ochre pieces are mostly composed of SiO_2_, Fe_2_O_3_, CaO, and K_2_O. They feature Zn, Ga, As, V, and SO_2_ as trace elements. The silica content varies notably across the samples, ranging from 5.5 to 14.1%, with generally higher values in MUH and ZSKVI samples (e.g., MUH-13, 14.0 ± 0.4%). Lower silica values in PRMII and certain ZSKV samples (e.g., PRMII-9e, 5.5 ± 0.1%) indicate a dominance of clay and iron-rich components over quartz inclusions. Iron content is consistently high across all samples, characteristic of ferruginous claystone and hematite-rich ochres. Fe_2_O_3_ values range from 40.1 to 47.2%, with notable concentrations in samples such as ZSKV-74 and ZSKVI-02. This elevated Fe_2_O_3_ content aligns with visual observations of red and yellow hues in the samples, suggesting a predominance of hematite and goethite as the key iron oxide phases. CaO values are variable but generally low (<2.0%), except for samples such as ZSKVI-04 (3.3 ± 0.2%), which aligns with the macroscopic identification of a calcitic crust. This suggests postdepositional calcite infiltration in certain fragments, a feature observed in microstructural analyses. K_2_O concentrations range between 0.5 and 2.0%, likely linked to clay minerals (illite or muscovite) present within the ferruginous matrix. TiO_2_ and MnO values remain consistently low across all samples, indicative of minimal titanium-bearing minerals or manganese inclusions. Among the trace elements, Zn concentrations are moderate, ranging from 220 to 430 parts per million (ppm), with higher values recorded in samples MUH-13 and Red Gully-2. Ga values are stable across samples (~19 to 23 ppm), supporting the clay-dominated mineral matrix. As values are generally low (<5 ppm), indicating minimal contamination from arsenopyrite or other As-bearing minerals. V concentrations, which are typically linked to iron oxides, vary between 140 and 400 ppm, with the highest values recorded in ZSKVI and PRMII samples. SO_2_ concentrations, as measured because of SEM coupled to energy-dispersive spectroscopy (SEM-EDS), remain low across most samples, indicating limited sulfide inclusions or weathering products such as gypsum. In summary, the elemental analysis confirms the ferruginous nature of the ochre fragments, characterized by high Fe_2_O_3_ content, low SiO_2_ in several samples, and minimal contamination from trace elements such as As. Variations in CaO reflect postdepositional processes (e.g., calcite crusting), while differences in Zn and V concentrations could relate to the geochemical signatures of raw material sources. However, the trace element values may be challenging to evaluate with precision, as the analyses were limited to 60 s. This short duration may have introduced matrix effects, where variations in mineral composition and sample heterogeneity could influence the detected concentrations of certain trace elements. The elevated iron oxide concentrations, combined with the observed textures and mineral inclusions, suggest deliberate selection of hematite-rich materials by Neanderthals for their vibrant red and yellow hues. In addition, the diversity in chemical composition indicates multiple source areas or processing techniques, reflecting a nuanced understanding of material properties. Overall, these results align with visual and microstructural analyses, highlighting the complex interplay between raw material availability, Neanderthal selection practices, and postdepositional processes affecting these ochre fragments.

### Multivariate analysis

**Fig. 9.**
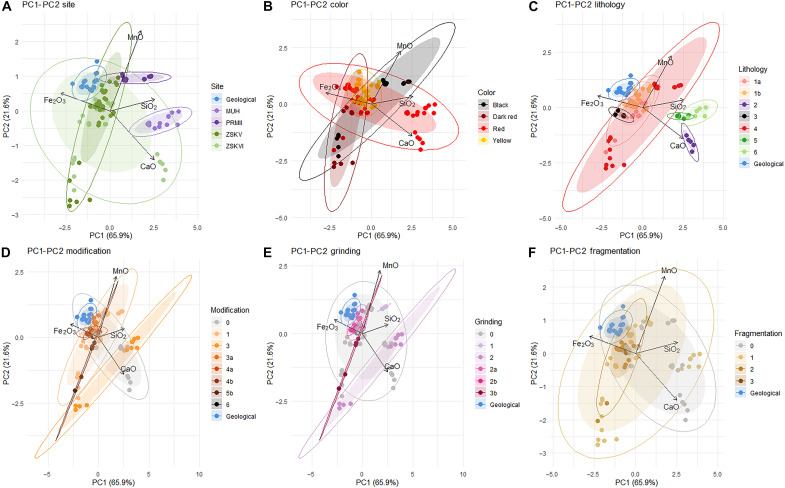
PCA. PCA of pXRF elemental data from 16 archaeological ochre pieces and 4 natural ochre samples from the Red Gully outcrop. The PCA is exploratory and aims to assess chemical variability among the analyzed specimens. The two samples from MUH are included for comparative purposes, as they derive from a Micoquian Mousterian context geographically distinct from the Crimean sites. The two darker pieces from PRMII were selected to expand the range of visual and compositional variation observed in the sample. The PCA is carried out according to site origin (**A**), color (**B**), texture (**C**), modification index (**D**), grinding index (**E**), and fragmentation index (**F**). Plots include 95% confidence ellipses.

To further explore compositional differences among the analyzed pieces, we conducted a principal components analysis (PCA) of the portable x-ray fluorescence (pXRF) elemental data (Fig. 9). The PCA is used here as an exploratory tool to visualize chemical variability across the subset of 16 archaeological samples and 4 natural ochres from the Red Gully outcrop. Because of the small sample size and nonrandom selection criteria—focused on preservation, color range, and signs of anthropogenic modification—these results are not statistically representative of full-site assemblages. Instead, the PCA highlights variation within the analyzed subset. The PCA (Fig. 9) successfully explains a substantial portion of the data variability, accounting for 87.8% of the variance. Breaking down the principal components, PC1, contributing to 68.1% of the variability, is primarily influenced by the concentrations of calcium (Ca), iron (Fe), and silicon (Si). This suggests that variations in these elements significantly contribute to the overall variability observed in the dataset. PC2, accounting for 19.7% of the variability, is primarily driven by the concentrations of calcium (Ca) and manganese (Mn). This indicates that changes in the levels of Ca and Mn play a significant role in the secondary principal component, contributing to the overall data variability but, to a lesser extent, compared to PC1.

When dissecting the variability in elemental composition by site (Fig. 9A), a pronounced similarity emerges in the ochre used at ZSKV and ZSKVI, with the sole exception being piece ZSKVI-04. This piece exhibits an elevated Ca content due to coating by a calcitic crust. Several pieces from ZSKV and ZSKVI closely resemble the composition of those from the natural outcrop. This similarity is not mirrored in the ochre from PRMII, which exhibits a higher concentration of Mn. The pieces from MUH stand out because of their lower Fe content and higher levels of Si and Ca. When delving into the PCA results based on the color of the ochre pieces (Fig. 9B), it becomes evident that blackish ochre, rich in Mn, is only used at PRMII. Dark-red ochre, characterized by higher Fe content, relatively lower levels of Si and Ca, and variable proportions of Mn, is commonly found at both ZSKV and ZSKVI. Red ochre, displaying greater compositional variability and richer in Si and Ca, is prevalent at these two sites and MUH. Yellow ochre, with a balanced composition of Fe, Mn, Si, and Ca, is also identified at ZSKV and ZSKVI. Both dark-red and yellow ochre exhibit compositional distributions that overlaps with those determined for the geological source. Breaking down PCA results by the lithology of the ochre pieces (Fig. 9C) produces results that echo to some extent differences observed in color. Groups 1 and 2, coinciding with the yellow ochre and the dark-red ochre richer in Mn, found at ZSKV and ZSKVI, are also those that show a composition similar to the geological source. Groups 5 and 6, rich in Si and Ca, are only found at MUH, and group 4 is found at ZSKVI. When coding the specimens in the PCA based on modification intensity (Fig. 9D), it appears that the more heavily modified pieces are those with the higher Fe content associated with a variable Mn content and that their compositional variability encompassed a portion of that collected at the natural source. Differentiating elemental contents based on the intensity of grinding and fragmentation applied to the ochre pieces (Fig. 9E-F) reveals a gradient in both cases. There is a transition from higher Si and Ca content toward higher Fe content, corresponding to an increase in the modification rate.

## DISCUSSION

The findings of this study enhance our understanding of the cognitive and cultural sophistication of Micoquian Neanderthals in Crimea and Neanderthal cultures more broadly. The stratigraphic provenance and chronological attribution of the ochre pieces reveal that Neanderthals in this region engaged in ochre use over a time span of up to 70,000 years. Ochre-bearing levels are documented from layer VI of ZSKV—attributed to MIS 5c (~100 to 90 ka)—through to layer II of ZSKVI, dated to ~33 to 36 ka cal B.P. While the archaeological record does not allow us to affirm continuous use across this interval, the recurrence of modified ochre pieces in multiple stratigraphic layers at different sites indicates that red rocks with coloring properties held enduring cultural significance within this regional Micoquian tradition. Multivariate analysis of ochre elemental composition, supported by optical and SEM-EDS inspection, indicates that Neanderthals exploited iron-rich rocks from outcrops located ~1.5 km from the ZSK sites—the first time this source has been directly sampled and matched to archaeological material—as well as from other, as yet unidentified, sources. Their selection and processing of ochre suggest a deep understanding of raw material properties. This interpretation is supported by the exploratory PCA, which illustrates chemical variability within the analyzed sample and highlights differences consistent with observed visual and textural traits. Neanderthals used various techniques—scraping, scoring, grinding, and flaking—adapting their methods to the physical characteristics of the material. Softer ochres were scraped and ground, while harder ones were flaked and pounded. These actions indicate a deliberate, methodical approach to ochre powder production and a refined chaîne opératoire. While we cannot entirely exclude the possibility that some variability in extraction techniques reflects experimentation or trial and error, the observed patterns suggest otherwise. Techniques appear to have been consistently selected on the basis of the raw material’s physical properties, with no evidence of inefficient or mismatched approaches—for example, no scraping of hard ochres or pounding of friable ones. In particular, the succession of technical actions observed on the crayon-like pieces (ZSKV-06 and ZSKV-07) follows a structured and functionally coherent sequence: initial shaping through scraping and grinding, followed by maintenance via knapping and regrinding to preserve a pointed morphology. This coherent chaîne opératoire indicates not only a goal beyond mere powder extraction but also a practical understanding of how to prolong tool use. These consistency is difficult to attribute to random experimentation and more plausibly reflects culturally transmitted knowledge and intentionality. ZSKV-07, although fragmentary, mirrors the technical sequence observed on the complete crayon (ZSKV-06), including shaping through scraping and grinding, followed by maintenance and reuse. Its transformation after breakage—groove incision and flake removal—suggests efforts to extend its use-life, reinforcing its interpretation as a curated tool rather than a by-product of pigment extraction.

Our findings do not imply that all Neanderthal ochre use in Crimea was symbolic nor that this behavior was continuous over tens of thousands of years. Rather, we show that specific pieces from well-dated Micoquian contexts exhibit characteristics—shaping, engraving, polish, and resharpening—best explained by symbolic use. Various techniques were applied depending on the physical properties of the material. The presence of different extraction techniques on the same objects suggests that individuals mastered a repertoire of coloring powder processing strategies. These behaviors, seen alongside engraved bones and curated objects from the same region, point to the presence of symbolic traditions in some Neanderthal groups and call for further research integrating technological, contextual, and experimental data. While most ochre pieces in our sample show evidence of modifications that could reflect either practical or symbolic applications, a subset of the assemblage—specifically pieces ZSKV-05, ZSKV-06, and ZSKV-07—exhibits features that are difficult to explain through utilitarian activities alone. Piece ZSKV-06 has a crayon-like form and shows repeated sharpening; ZSKV-07 may represent a fragmented crayon; and ZSKV-05 bears deliberate, subparallel engraved lines along with surface smoothing and polish on prominent areas, suggesting careful curation and reuse. We acknowledge that the presence of pointed ochre pieces does not automatically imply symbolic use, as demonstrated by Wadley (*74*) replication experiments at Sibudu Cave. In those studies, ochre nodules ground to produce powder sometimes developed crayon-like shapes as incidental by-products of grinding, lacking distinct use-wear patterns on the tips. Analysis of these experimental pieces revealed no difference between the wear on the lateral facets and the tip and no application of distinct complementary techniques for maintaining the latter. By contrast, Crimean ochres, such as ZSKV-06 and ZSKV-07, exhibit a palimpsest of different technical actions, suggesting deliberate gross manufacturing by scarping, shaping by grinding, and subsequent knapping and grinding to secure maintenance of a pointed tip.

The shaping of ochre fragments into crayon-like tools through scraping and grinding, along with wear and maintenance traces from knapping and regrinding to keep them pointed, suggests that these implements were used to create red and yellow marks or designs on various surfaces, including skin, clothing, bags, or stone. The homogeneous application of ochre to these items—for example, to tan hides or coat surfaces—would not have required pointed tools; rubbing raw ochre or smearing an ochre-rich liquid compound would have sufficed. In contrast, the deliberate shaping and resharpening of ochre crayons indicate that their tips were specifically used to produce linear marks. This interpretation aligns with evidence from Middle Stone Age contexts, such as the silcrete fragment from Blombos Cave bearing a cross-hatched pattern interpreted as a deliberate design drawn using an ochre crayon (*75*). Experimental replication confirmed that these marks were most plausibly produced by dragging the tip of a shaped ochre piece across a stone surface, reinforcing the plausibility of similar marking behavior at the Crimean Micoquian sites. One ochre piece featuring curved, subparallel engraved lines, displays a degree of gestural consistency that surpasses utilitarian scraping for ochre powder extraction. This evidence suggests intentionality beyond practical use. Along the production of ochre crayons, the deliberate nature of these engravings aligns with probable nonutilitarian functions.

Interpretive caution is, of course, warranted when evaluating the use of mineral colorants, given their multifunctionality. Ethological examples, such as the deliberate staining of plumage by bearded vultures (*Gypaetus barbatus*) in ferruginous pools, show that even nonhuman animals engage in behaviors involving color application for reasons that remain debated—ranging from parasite control to status signaling (*76*). In archaeology, similar ambiguities exist: The presence of ochre or manganese oxides does not inherently imply symbolic behavior. Interpretations must be grounded in technological and contextual evidence.

In our case, the deliberate shaping and reuse of crayons, the engraved motifs, and the evidence for curated tools collectively support the conclusion that at least some ochre materials were involved in symbolic activities. These objects and the markings they produced likely played roles in communication, identity expression, and intergenerational knowledge transmission. The curated nature of the ochre fragments further supports this interpretation, suggesting that they were preserved, transported, and reused—behaviors that reflect both planning and cultural investment.

Recent research has shown that many radiocarbon dates from Micoquian sites in Crimea, including ZSKV and ZSKVI, may be significantly underestimated because of contamination issues (*77*). This implies that the true age of these sites—and of the ochre documented here—may extend 10,000 radiocarbon years further back in time than previously thought. If so, then Neanderthals in Crimea engaged in ochre use for an even longer period, reinforcing the case that these activities reflect persistent cultural traditions rather than short-term adaptations near the end of Neanderthal history. This revised chronology also raises questions about potential interactions with *H. sapiens* populations and the idea of Crimea as a Neanderthal refugium at the Middle-Upper Paleolithic transition. Establishing a more precise timeline through refined radiocarbon dating techniques, such as single amino acid dating, will be essential to reassessing the persistence and evolution of symbolic behaviors in Neanderthal populations.

Additional evidence of symbolic practices in the Crimean region lends supports to the interpretation of Neanderthal engagement in symbolic practices. The engraved cortex from the Kiik-Koba site (*78*) and the decorated raven bone from ZSKVI (*79*) suggest that Neanderthals in the region actively created and transmitted a symbolic material culture, reflecting a cognitive complexity previously underestimated.

These behaviors find parallels in contemporary Middle Stone Age contexts from southern Africa. At Blombos Cave, Klasies, and Klein Kliphuis, ochres were processed with techniques similar to those observed in Crimea (*80*, *81*), engraved with abstract motifs (*31*) (including subparallel lines), and shaped into crayons to mark stone flakes (*32*). Technological parallels also extend to bifacial tool production: Both the Micoquian and the Still Bay traditions exhibit advanced craftsmanship in bifacial tool production, pointing not only to innovations but also to symbolic or communicative material culture. These cross-continental similarities challenge longstanding assumptions about Neanderthal cognition. Shared cognitive capabilities among early human populations across regions contribute to the ongoing reassessment of Neanderthal behavior and cognition. Traditionally viewed as lacking the cognitive flexibility and symbolic capacity of anatomically modern humans, the Neanderthals of Crimea demonstrate the opposite: They engaged in cultural practices that were not merely adaptive but deeply meaningful. Their sophisticated use of ochre is one facet of their complex cultural life, challenging previous assumptions and underscoring their similarity to contemporary human populations in Africa. Similar to their *H. sapiens* contemporaries, Crimean Micoquian Neanderthals actively shaped what we may call an epistemic niche, i.e. a cultural and conceptual space structured through shared knowledge, material engagement, and symbolic communication. The creation of this niche, as well as the cultural strategies it entailed, appears to have transcended “species” barriers, reflecting deep cognitive convergences. Last, the use of ochre in this region also provides important insights into the regionalization of Neanderthal cultures. Rocks with coloring properties are not used uniformly across all Neanderthal populations. In some regions, black materials—typically manganese-rich rocks—were favored; in others, red and yellow ochres played a prominent role. This variability suggests different cultural trajectories, possibly involving community-level traditions, long-distance exchanges, or localized innovation. The presence of ochre in the Crimean Micoquian strengthens the view that continuity in the use of this lithic technology reflects an enduring cultural trajectory—one in which symbolic and technological behaviors were deeply intertwined.

## MATERIALS AND METHODS

The 16 archaeological ochre pieces analyzed in this study were selected on the basis of their unambiguous identification as rocks with coloring properties through macroscopic and microscopic inspection and the presence or potential presence of anthropogenic modifications. The total figure of 291 ochre or ochre-stained items reported in the literature for Crimean Middle Paleolithic sites includes numerous fragments of uncertain status—such as reddish clasts—which were not systematically verified. In addition, several pieces were excluded from analysis due to their small size or thinness, which rendered them incompatible with reliable pXRF analysis. Some ochre fragments identified more recently, after the end of the collaborative phase of this project, could not be studied because of current limitations on access to collections in Ukraine. All coloring materials were photographed for documentation with a Canon G7X camera incorporating multiple aspherical elements to enhance image quality by reducing aberrations and distortions. Photos were used to document both the general aspect of each piece and the traces of modifications present on them. Observations were also carried out with a motorized Leica Z6 APOA, equipped with a DFC420 digital camera linked to a LAS Montage and Leica Map DCM 3D computer software. We produced a detailed description for each ochre piece, emphasizing specific and noteworthy characteristics. We also recorded contextual, technological, and morphometric information about all ochre pieces including site, archaeological layer in which the piece was found, length, width, thickness, and weight of complete objects, raw material type, color, and morphology of the piece (slab, pebble, nodule, chunk, flake, crayon, and irregular). Anthropogenic modifications (scraping, grinding, smoothing, flaking, percussion marks, and fracture) were identified macro- and microscopically using established methodologies from existing literature (*33*, *82*, *83*). For each sample, the presence (1) or absence (0) of each modification type was systematically recorded (Table 1). On the basis of these observations, three indices were developed: the grinding index, fragmentation index, and modification index. The grinding index was calculated as the sum of the presence values for scraping, grinding, and smoothing. The fragmentation index was calculated as the sum of the presence values for flaking, percussion marks, and fractures. The modification index was calculated as the sum of the presence values for all recorded anthropogenic modifications. This systematic approach facilitates the quantitative comparison of different types of modifications across samples.

### Portable x-ray fluorescence spectrometry

The elemental composition of both archaeological and natural samples was noninvasively assessed using pXRF spectrometry. Fourteen archaeological and four geological samples were analyzed using an AMETEK portable SPECTRO xSORT XRF at the UMR 5199, PACEA (De la Préhistoire à l’Actuel: Culture, Environment et Anthropologie), University of Bordeaux, France. The XRF spectrometer is equipped with a silicon drift detector (SDD), a low-power W x-ray tube with an excitation source of 40 kV and an x-ray beam of 8 mm. Careful attention was given to positioning the piece in close proximity to the detector, ensuring that only flat, clean areas free from sediment, concretions, or other interfering materials were analyzed. Three to eight measurements were taken on each piece with a spectra acquisition time of 60 s. The spectrometer is internally calibrated by an automated measure of the elemental content of a standard metal shutter. A supplementary calibration (*40*, *84*), based on the Lucas-Tooth and Price (*85*) methodology, was applied. This calibration, developed with X-LabPro software (AMETEK, Berwyn, USA), adjusts the mass attenuation coefficient and calibration slopes for seven major and trace elements using certified standards with variable content of iron oxide and reference samples analyzed by inductively coupled plasma optical emission spectrometry and inductively coupled plasma mass spectrometry at the Service d’Analyse des Roches et Minéraux, Nancy, France (*50*). Three pressed pellets of international standards were analyzed to evaluate eventual measurements drift: DRN (diorite), MicaMg (phlogopite), and SARM69 (ceramic). Each standard was analyzed five times. Results of these reference measurements are presented in table S5.

### Scanning electron microscopy coupled to energy-dispersive spectroscopy

Microscopic observations of selected representative pieces were carried out with a SEM-EDS. Selected samples were cleaned with compressed air. Observations were performed with two different equipment, a FEI Quanta 200 coupled with an SDD-EDAX detector, located at the Bordeaux Imaging Center, University of Bordeaux, and a Zeiss Evo 15 SEM coupled to an Oxford energy-dispersive spectrometer located at the UMR 5199, PACEA, University of Bordeaux. Four archaeological samples were analyzed with the Fei Quanta 200, and six archaeological samples and one geological sample were analyzed with the Evo 15. SEM observations carried out with the Fei Quanta 200 were conducted under a low vacuum mode using an accelerating voltage of 15 kV. Similar magnifications (×1450, ×3000, and ×6000) were used for the EDS analyses of each sample, and the working distance was kept constant (~10 mm). Acquisition time was set up to 100 s for each EDS spectrum. SEM analyses, producing backscattered electron images and elemental analyses were carried out with the Zeiss Evo 15 and performed in low vacuum with a pressure of 30 Pa and an accelerating voltage of 20 kV at a working distance of 8.5 mm.

### Statistical analysis

PCA, commonly used in ochre studies (*23*, *86*–*88*), was applied to pXRF data to investigate the compositional variations of the coloring materials. The PCA was conducted on elemental data from 16 archaeological ochre pieces and 4 natural samples collected from the Red Gully outcrop near ZSK. The PCA was exploratory in nature and intended to assess compositional variability among the analyzed specimens. It was not designed to characterize the full ochre assemblages from each site or to support statistically representative comparisons across sites. Measurements were not averaged before the PCA analysis, but intrasample comparisons were conducted to verify data consistency. With the exception of piece ZSKVI-04, measurements performed on areas affected by postdepositional phenomena (calcitic crust, ink...) were not included in statistical analyses. Only elements that could be reliably measured as major and minor elements in a majority of the samples, i.e., not below the limit of detection, were used for multivariate analysis. These elements include Si, Ca, K, Ti, Mn, and Fe. To normalize the magnitude differences between elements, a log-Fe transformation was applied to the data (*23*, *87*–*89*). The Fe content underwent transformation through a logarithmic function. This allowed accounting for Fe variability while ensuring a similar weight to all variables. Variables reduction was carried out in an empirical manner by removing elements correlated to other elements and giving minor contributions to the normalized dataset variability. Si, Ca, Mn, and Fe were the only elements used for subsequent statistical analyses. All statistical analyses were performed with the R software version 4.2.3 (R Core Team, 2022) (*90*) and the “ggplot2” (*91*), “FactoMineR” (*92*), “factoextra” (*93*), “ggpubr” (*94*), and “cowplot” (*95*) packages. The reproducible R script is presented in texts S1 and S2.

## Supplementary Materials

### This PDF file includes:

Texts S1 and S2 Figs. S1 and S2 Tables S1 to S6 References
