# Supplementary material for: Evidence for symbolic use of ochre by Micoquian Neanderthals in Crimea
Source: Sci Adv. 2025 Oct 29;11(44):eadx4722. doi: 10.1126/sciadv.adx4722 (PMC12571073; doi:10.1126/sciadv.adx4722)
Supplement: Supplementary file 1 — Texts S1 and S2 Figs. S1 and S2 Tables S1 to S6 References [file sciadv.adx4722_sm.pdf]

Science Advances
AAAS

Supplementary Materials for

Evidence for symbolic use of ochre by Micoquian Neanderthals in Crimea

Francesco d'Errico *et al.*

Corresponding author: Francesco d'Errico, francesco.derrico@u-bordeaux.fr

Sci. Adv. **11**, eadx4722 (2025)
DOI: 10.1126/sciadv.adx4722

This PDF file includes:

Texts S1 and S2
Figs. S1 and S2
Tables S1 to S6
References

Supplementary Text S1

Provenance

The items analyzed in this study originate from several multi-layered Middle Paleolithic sites in Crimea and Ukraine. Material from Zaskalnaya V and Zaskalnaya VI (Kolosovskaya), as well as Prolom II, was collected during systematic archaeological excavations conducted in the 1970s and 1980s by the Crimean Paleolithic Expedition of the Institute of Archaeology, Academy of Sciences of the Ukrainian SSR, under the direction of Y.G. Kolosov. Subsequent revision and identification of these items within the assemblages took place between 1985 and 2010 and were carried out by V.N. Stepanchuk. Material from the natural ochre deposit near Krasnaya Balka (Crimea) was collected in 2005 during fieldwork directed by V.N. Stepanchuk as part of the Crimean Paleolithic Expedition of the Institute of Archaeology, National Academy of Sciences of Ukraine. Artifacts from the Mukhovets Paleolithic site were recovered in 2000 during fieldwork conducted by the Romny Paleolithic Expedition of the Institute of Archaeology, National Academy of Sciences of Ukraine, led by Y.V. Kukharchuk. The artefactual status of the objects from Zaskalnaya V and VI, Prolom II, Mukhovets, and the Krasnaya Balka ochre locality was confirmed, upon close analysis, by the five of us (FD, GM, AP, AM, VS). While the objects themselves were not directly dated, the archaeological layers in which they were found have been dated using radiocarbon and thermoluminescence (TL) methods. Chronological attribution is further supported through the integration of geological, palaeontological, and palynological analyses. All materials from the archaeological sites of Zaskalnaya V and VI, Prolom II, and Mukhovets are currently housed at the Institute of Archaeology, Department of Stone Age Archaeology, Kyiv, Ukraine. The geological sample from Krasnaya Balka is curated at the same institution. These collections are available for scientific study upon submission of a formal request and justification of research aims.

Archaeological context

Stepanchuk (*70*, *96*) recognizes the presence of two types of bifacial industries in Crimea: the Micoquian, characterized by bifacial backed knives, and the para-Micoquian rich in bifacial leaf points. This article focuses on ochre from three Micoquian sites of the Crimean Peninsula: Zaskalnaya V, Zaskalnaya VI (Kolosovskaya) and Prolom II. The last site is located in eastern Crimea at 6.5 km from Zaskalnaya V and Zaskalnaya VI. All these sites are attributed to the Micoquian and in particular to its Ak-Kaya variant (*68*, *69*, *96*). For comparative purposes and their potential relevance in discussing the symbolic use of ochre, we also included two ochre

pieces recovered from the Mousterian site of Mukhovets, located in the Sumy Oblast near the eponymous village in northern Ukraine.

Zaskalnaya V

The Middle Paleolithic site of Zaskalnaya V (45°06'N; 34°36'E) is a collapsed rock shelter situated at the base of a limestone cliff, close to the village of Belaya Skala, north of Belogorsk in the east of the Crimean Peninsula. The site is 189 m above the sea level and 60 m above the Bijuk Karasu River. Zaskalnaya V bears a long Middle Paleolithic stratigraphic sequence reported thus far from a rock shelter in Eastern Europe and has garnered significant renown due to the richness of its archaeological assemblages and the discovery of Neanderthal remains. Zaskalnaya V was discovered by Petrun in 1964 and excavated by Kolosov between 1969 and 1994. The main excavation extended over a surface of 32.5 m^2 and reached the bedrock at a depth of about 4.5 m. Several test pits were also dug by Kolosov on the slope below the site. Of particular interest is the so-called *Zaskalnaya V Trench*, located 15 m down the slope of main Zaskalnaya V site. Zaskalnaya V trench sediments cover the buried cave site of Alyoshin Grotto (*97*) and contain two levels of artefacts, fauna and Neanderthal remains likely slid down from layers II-IV of Zaskalnaya V. A revaluation of the Zaskalnaya V stratigraphy and associated archaeological assemblages was undertaken by Kolosov and Stepanchuk in 1997. This was followed by a new excavation directed by Chabai in 2012 and 2013 over a surface of 4 m^2 (*98*).

Stratigraphic and archaeological evidence from the Kolosov excavation were extensively analyzed and published (*68*, *97*, *99–104*). These studies led to the identification, from the top to the bottom of the sequence, of eight cultural layers: I, Ia, II, III, IIIa, IV, V, VI, each of them between 10 to 50 cm thick, separated from one another by sterile layers of similar thickness. Lowermost layer VI and V are attributed by Kolosov and colleagues to MIS 5c. Layers IV to I would cover the period between MIS 5a and MIS 3 (table S2). Lithic industries from all layers are attributed by Kolosov and Stepanchuk (*105*) to a local Micoquian of Ak-Kaya type. Differences between layers, interpreted by Gladilin (*106*) as indicating that layers I and V should be rather attributed to the Kiik Koba culture are now interpreted as primarily due to different degrees of resharpening depending on the varying distance of the site from raw material sources (*107*). A fragment of the skull of a 5-6-year-old child was discovered in Layer V (*70*). A fragment of the occipital bone from a 25-26-year-old Neanderthal individual and a fragment of a human metacarpal were also found in these layers (*102*, *108*).

Chabai excavation identified 23 lithological layers and 87 archaeological levels, some of which witnessing phases of stratigraphic stability and rapid accumulation, other episodes of erosion and bioturbation indicating a number of stratigraphic discontinuities. The new excavation confirmed the absence of Upper Paleolithic layers and the cultural attribution of lithic assemblages from all layers to the Crimean Micoquian, albeit with differences in the frequency and size of the main tool types. The Zaskalnaya V sequence was submitted to four dating campaigns using AMS measurements on bone (*101, 109*), AMS bone and charcoal (*98*) and ESR on teeth (*110*). Although inversions are observed in the ages obtained by 14C and ESR and between 14C ages obtained with different sample pretreatment methods (table S2), the resulting pattern is consistent with the attribution of an age of c. 42-46 ky cal BP to the top of Kolosov cultural unit III and the bottom of this cultural unit II, cultural unit I being contemporaneous or older than c. 33-35 ky cal BP, and cultural unit IV and lower being older than 47 ky cal. BP.

The presence of ochre and ochre-stained items were first signalled by Kolosov in layers IV and II (*69*, *70 73*). It is now recorded in six (I, II, III, IV, V, VI) of the eight layers identified in the main excavation and in the *Zaskalnaya V Trench*. In a recent work Stepanchuk (*73*) describes a fragment of a pebble from the Trench bearing a red and yellow spot associated with longitudinal striations interpreted as possibly resulting from the use of the pebble as a *retouchoir*. The seven ochre pieces from Zaskalanaya V analyzed in the present study come from Layers II, V, VI and the Trench (table S3). The attribution of the lowermost layers V and VI to MIS 5c (~100–90 ka) is supported by their stratigraphic position beneath layers dated by radiocarbon and ESR to >47 ka cal BP, and by geomorphological and paleoclimatic correlations with regional erosion surfaces and loess-paleosol sequences (*103*, *111–113*). Together with younger ochre-bearing layers at Zaskalnaya VI, this framework supports a conservative estimate of pigment use spanning as much as 70,000 years in the Crimean Middle Paleolithic.

Zaskalnaya VI (Kolosovskaya)

Zaskalnaya VI, also known as Kolosovskaya or the site of Kolosov—named after the archaeologist who discovered it in 1969 and conducted extensive research on the Crimean Middle Paleolithic— is situated at 45°06'N, 34°36'E near Vishennoye village in the Belogorsk District, along the Biyuk-Karasu river, in Crimea. The shelter faces south and lies at an elevation of 205 m above sea level, 60 m above the river, about 35 km from the current coastline. The site underwent intensive excavations from 1969-1975, 1977-1978, 1981-1985,

and 2005, covering an area of 78 m^2 and reaching a depth of approximately 3 m (*69, 100, 114*). The stratigraphy includes seven Middle Paleolithic layers and sublayers, named from the bottom to the top: VI, V, IV, IIIa, III, II, and I. Between 1969 and 1985, only retouched artifacts, cores, and identifiable large bones were spatially recorded. All other objects were assigned to a cultural layer, a specific square meter, and the upper and lower depth of that layer within the square meter. In the excavation conducted in 2005 by one of us (VS) all archaeological remains larger than 2 cm were spatially plotted, and sediment was systematically sieved with a 1 mm mesh. The lithic assemblages from all layers are attributed to the Micoquian of Ak-Kaya type, with no Upper Paleolithic layers or artifacts found on site (*105*). This industry includes non-Levallois centripetal and sub-parallel non-volumetric core reduction and bifacial shaping, constituting up to 30% of the tools. The tool assemblage comprises foliated points, side scrapers, and foliated backed knives. Fireplaces and pits identified in layers II, III, IIIa and IV support the site's stratigraphic integrity (*105*). The available seventeen AMS 14C ages on bone collagen attribute an age of 26-29 ky cal BP to layer I, 33-36 ky cal BP to layer II, 38-43 ky cal BP to layer III, 36 - 46 ky cal BP to layer IIIa and an age of over 47 ky cal BP to layer IV (table S2). The relatively recent age of the layers I-III, compared to those of the initial Upper Paleolithic in other European regions, has led authors to propose the hypothesis that this region of Crimea, if not the whole peninsula, acted as a refuge for the last Neanderthals of Eastern Europe. Numerous Neanderthal human remains of all ages, from newborn to adults, were found in layers IIIa, III, II and are particularly abundant in the first two layers (*69, 102*, *104*, *115*–*117*). A possible burial pit associated with the remains of three adolescents was identified in layer III (*69*, *71*, *115*). Ochre and ochre-stained items were found in layers I (n=1), II (n=16), III (n=23), IIIa (n=2), IV (n=13), V (n=2), VI (n=1). The five ochre pieces from Zaskalnaya VI analyzed in the present study come from Layers I/II, II, and IIIa (table S3).

Prolom II

Prolom II is a multilayered site located in the Belogorsk District, Crimea, on the left bank of the Kuchuk-Karasu River and close to the homonymous village, at 45°06'N, 34°42'E. The site is about 6,5 km as the crow flies from the Zaskalnaya V and VI. The stratigraphic sequence is situated at the base of a rock shelter, which was once a cave formed within a massif of nummulite limestone, now collapsed. The shelter faces east at an elevation of 205 m above sea level and 15 m above the river. Only the western portion of the shelter roof is preserved. The total investigated area covers a surface of 59 m^2. Discovered by Kolosov in 1973, it was excavated in 1977, 1981, 1982, 1985, and 1997 (*69, 101, 118*) . The 1.7 m thick deposit features

eight geological and four cultural layers, all defined as Micoquian (*70*, *118*). More than 30 species of mammals were identified in the cultural layers of the site. Among the large mammal species, the saiga dominates (53%), followed by horse (11%), bison (3%), cave hyena (6%) and cave bear (2.4%) (*118*). Enloe and colleagues (*119*) propose that the shelter was alternately occupied by humans and cave hyenas. Bone fragments and teeth bearing traces of modification interpreted as demonstrating non-utilitarian activities were identified in the cultural layers (*119*). Isolated human hand phalanxes were found in Middle Paleolithic layers I and II (*70*). Radiocarbon ages chronologically attribute the sequence as follows: layer I (22900 ± 300 Ki-10746; 22650 ± 100 GrA-5445; 22800 ± 600 Ki-10895; 24550 ± 300 Ki-10745), layer II (28100 ± 350 Ki-10617), layer III (41600 ± 800 Ki-10611). Stepanchuck and colleagues (*71*) report the discovery of 44 possible mineral pigments from the site, most of them from the excavations conducted in 1981 (n=25) and 1982 (n=16). They notice that the higher number of ochre finds plotted in the excavation plans may be due to the fact that some fragile pieces could not be recovered. The stratigraphic distribution of the recovered pieces show they were almost exclusively present in the three upper layers and particularly abundant in layers 1 and 3. The ochre predominantly occurs in the form of small complete or broken pebbles and flakes. According to Stepanchuck, a few angular ochre pieces occur in several layers but are concentrated in layer 1. The abundance of pebbles suggests the raw material was mainly but not exclusively collected in alluvial deposits, probably those of the close Kuchuk-Karasu River. Most ochre pieces bear evidence of deliberate crushing, flaking and grinding but not scraping. The anthropogenic origin of individual striations is less clear considering that many pieces are soft and may have been damaged by transport, trampling and post-depositional processes. Interestingly, red ochre is only found in the three upper layers and is particularly abundant in layer I, a layer in which yellow ochre is absent (*71*).

Mukhovets

Mukhovets is an open-air site located in the Sumy region of northeastern Ukraine, approximately 650 km north of the Crimean Peninsula. The site was identified during pilot testing by Yu.V. Kukharchuk in the early 2000s (*120*), which revealed two distinct artefact-bearing horizons. The earlier horizon is attributed to the Kaydaky complex and is dated to Marine Isotope Stage (MIS) 5e. This complex forms part of the broader loess–paleosol sequence of Ukraine, typically underlying the Pryluky complex (MIS 5c–a). Both soil horizons represent periods of soil formation during warmer, more humid climatic conditions. The

Kaydaky horizon, in particular, is associated with forested landscapes, as suggested by palaeobotanical analyses, and reflects Neanderthal occupation during favorable environmental phases. At Mukhovets, two ochre pieces were recovered beneath the archaeological layer and are presumed to be associated with this cultural horizon. No additional ochre fragments were identified. The later horizon at Mukhovets corresponds to the Pryluky complex (MIS 5c–a), which is broadly contemporaneous with Layer V of Zaskalnaya V and Layer IV of Zaskalnaya VI in Crimea (*121*). While the stratigraphic integrity of the site appears reliable, the lithic material is sparse and not yet diagnostic. Some indications suggest that the assemblages may be attributed to the Denticulate Mousterian, though this remains to be confirmed. As archaeological research at Mukhovets is still in its early stages, the available material remains limited. Nonetheless, the two ochre fragments included in this study provide a rare opportunity to examine mineral colorant use in a region and cultural context that remains underrepresented in studies of Neanderthal behavior.

Supplementary Text 2

Script R

```
# Load required libraries
library(ggplot2)
library(FactoMineR)
library(factoextra)
library(ggrepel)
library(ggpubr)

# Load data
data_path <- "C:/Users/ZSK /Desktop/Data-supplementary ZSK.csv"
Data <- read.csv(data_path, row.names = 1)
Data$Sample <- factor(Data$Sample)
Data$Site <- factor(Data$Site)
Data$Lithology <- factor(Data$Lithology)
Data$Modification.index <- factor(Data$Modification.index)
Data$Grinding.index <- factor(Data$Grinding.index)
Data$Fragmentation.index <- factor(Data$Fragmentation.index)

# Perform PCA
pca1 <- PCA(Data[, c(10,11,13,15)], graph = FALSE)
# Calculate percentage of variance for the axes
percent_var <- round(pca1$eig[1:2, 2], 1)
```

```
# Prepare data for individuals
pca_data <- data.frame(
  PC1 = pca1$ind$coord[, 1],
  PC2 = pca1$ind$coord[, 2],
  Sample = Data$Sample,
  Site = Data$Site,
  Color = Data$Color,
  Modification = Data$Modification.index,
  Grinding = Data$Grinding.index,
  Fragmentation = Data$Fragmentation.index,
  Lithology = Data$Lithology
)

# Prepare data for variables (arrows)
scaling_factor <- 3
var_data <- data.frame(
  PC1 = pca1$var$coord[, 1] * scaling_factor,
  PC2 = pca1$var$coord[, 2] * scaling_factor,
  Variable = rownames(pca1$var$coord)
)

# Define a custom color palette for each group
color_palette_lithology <- c("1a" = "#fb9a99", "1b" = "#fdbf6f", "2" = "#6a3d9a", "3" = "#000000",
                             "4" = "#e31a1c", "5" = "#33a02c", "6" = "#b2df8a", "Geological" = "#4A90E2")
color_palette_site <- c("Geological" = "#4A90E2", "MUH" = "#9B77D4", "PRMII" = "#6A3D9B",
                        "ZSK-V" = "#6B8E23", "ZSK-VI" = "#A8D08D")
color_palette_color <- c("Black" = "#000000", "Red" = "#FF0000", "Dark Red" = "#8B0000",
                         "Yellow" = "#FFCC00", "Geological" = "#4A90E2")
color_palette_modification <- c("0" = "#bdbdbd", "1" = "#fdbb84", "2" = "#fec44f", "3" = "#fe9929",
                                "3a" = "#ec7014", "4a" = "#cc4c02", "4b" = "#993404", "5b" = "#662506",
                                "6" = "#000000", "Geological" = "#4A90E2")
color_palette_grinding <- c("0" = "#bdbdbd", "1" = "#d4b9da", "2" = "#c994c7", "2a" = "#df65b0",
                            "2b" = "#e7298a", "3a" = "#ce1256", "3b" = "#980043", "Geological" = "#4A90E2")
color_palette_fragmentation <- c("0" = "#bdbdbd", "1" = "#dfc27d", "2" = "#bf812d", "3" =
"#8c510a",
                                 "Geological" = "#4A90E2")

# Function to generate PCA plots with customized colors
generate_pca_plot <- function(data, variables, color_group, title, color_palette) {
  ggplot() +
    geom_point(data = data, aes(x = PC1, y = PC2, color = .data[[color_group]]), size = 3) +
    scale_color_manual(values = color_palette) +
    scale_fill_manual(values = color_palette) +
    geom_segment(data = variables, aes(x = 0, y = 0, xend = PC1, yend = PC2),
                 arrow = arrow(length = unit(0.2, "cm")), color = "black") +
    geom_text_repel(data = variables, aes(x = PC1, y = PC2, label = Variable),
                    color = "black", size = 4, max.overlaps = 10) +
```

```r
  stat_ellipse(data = data, aes(x = PC1, y = PC2, group = .data[[color_group]], color =
.data[[color_group]]),
               type = "norm", level = 0.95) +
  stat_ellipse(data = data, geom = "polygon",
               aes(x = PC1, y = PC2, group = .data[[color_group]], fill = .data[[color_group]]),
               level = 0.90, alpha = 0.2) +
  theme_minimal() +
  labs(
    title = title,
    x = paste0("PC1 (", percent_var[1], "%)"),
    y = paste0("PC2 (", percent_var[2], "%)")
  ) +
  theme(legend.position = "right")
}

# Generate PCA plots with specific color palettes
pca_plot1 <- generate_pca_plot(pca_data, var_data, "Site", "PCA 1-2 Site", color_palette_site)
pca_plot2 <- generate_pca_plot(pca_data, var_data, "Color", "PCA 1-2 Color", color_palette_color)
pca_plot3 <- generate_pca_plot(pca_data, var_data, "Lithology", "PCA 1-2 Lithology",
color_palette_lithology)
pca_plot4 <- generate_pca_plot(pca_data, var_data, "Modification", "PCA 1-2 Modification",
color_palette_modification)
pca_plot5 <- generate_pca_plot(pca_data, var_data, "Grinding", "PCA 1-2 Grinding",
color_palette_grinding)
pca_plot6 <- generate_pca_plot(pca_data, var_data, "Fragmentation", "PCA 1-2 Fragmentation",
color_palette_fragmentation)

# Combine the plots into a grid
x11()
ggarrange(pca_plot1, pca_plot2, pca_plot3, pca_plot4, pca_plot5, pca_plot6,
          labels = c("A", "B", "C", "D", "E", "F"),
          ncol = 3, nrow = 2)
```

Figs. S1 to S2

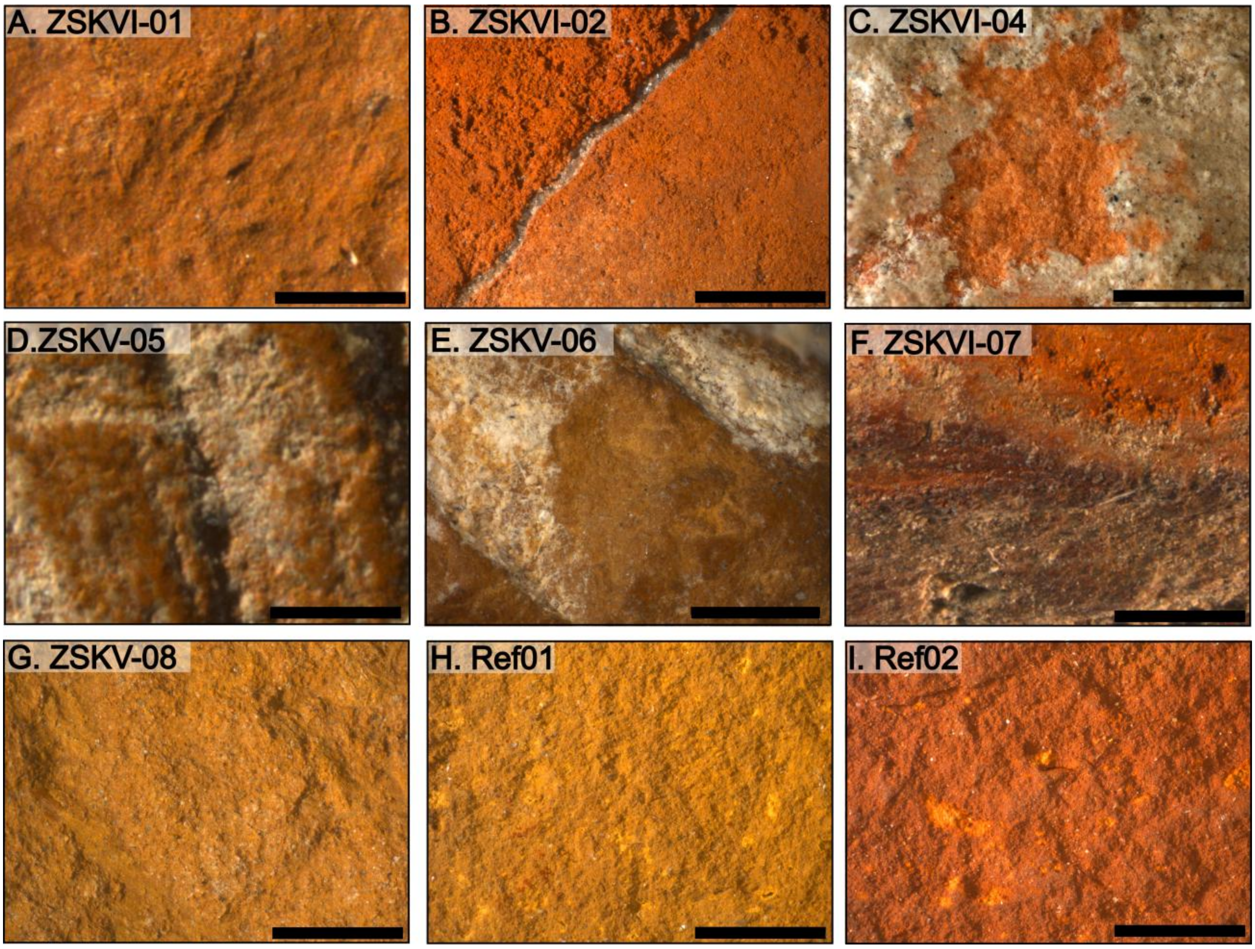

Fig. S1.

Microscopic views of ochre pieces. Microscopic photographs of selected coloring materials from ZSKVI, ZSK V and Red Gully (H. Ref01 and I. Ref02). All black bars are = 1 mm.

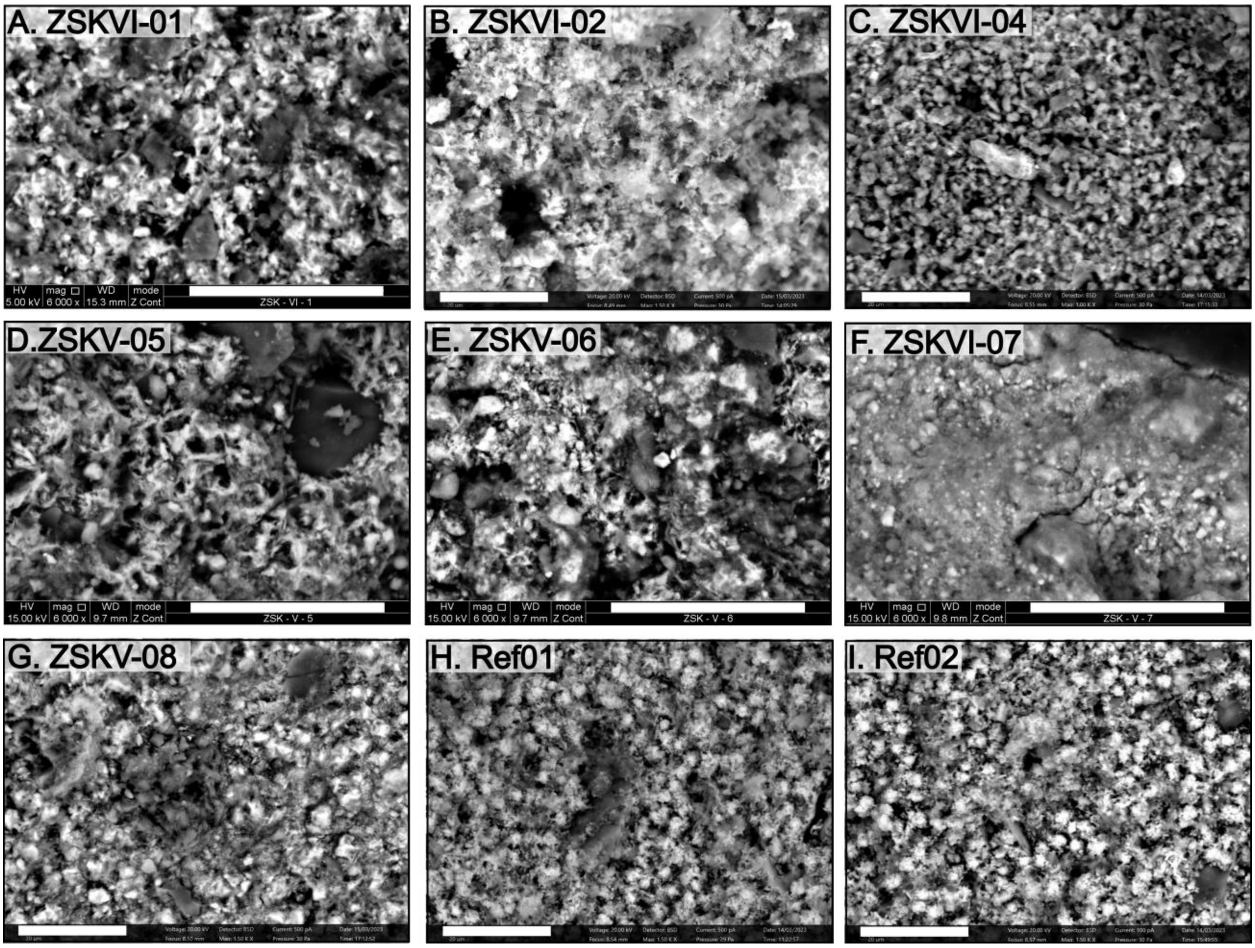

Fig. S2.

SEM Images of ochre pieces. Scanning electron microscopy images of selected coloring materials from ZSKVI, ZSK V and Red Gully. All white bars are = 20 µm.

Tables S1 to S6

Site	ID	Country	Culture	Layer	Colour				Analysis	Modifications	Conf.	Refs
					B	R	Y	W				
Ambrona	1	Spain	Late Ach.	Unknown		?				Indet	Low	(35, 122, 123)
Terra Amata	2	France	Late Ach.	C1a, C1b		X	X		El, Mi	Sm, Sc	Med	(34, 122–125)
Isernia la Pineta	3	Italy	Acheulean	Unknown		X				Indet	Low	126
Dashtadem 3	4	Armenia	Late Ach.	Unknown		X				Indet	Low	56
Hungsi V	5	India	Late Ach.	Unknown		X				Gr ?	Low	(57, 127, 128)
Cueva de Ardales	6	Spain	Mou	MP	X	X	X		El, Mi	Indet	Low	129
Cueva de los Aviones	7	Spain	Mou	II-III		X			El	Indet	High	47
Cueva Antón	8	Spain	Mou	I-K		X			Mi	Pulv, Paint	High	47
Cueva del Castillo	9	Spain	Mou	18		X		X	El	Sc, Gr	Med	130
La Quina	10	France	Mou	Upper Mousterian	X					Indet	Med	46
Tabaterie	11	France	Mou	Unknwon	X					Indet	Med	(42, 46)
Station du Bonhomme	12	France	Mou	Upper	X	X	X			Sc, Gr	Med	131
La Ferrassie	13	France	Mou	Unknown		X	X			Indet	Med	132
La Micoque	14	France	Mou	3			X			Indet	Med	46
Le Moustier	15	France	Mou	B-K	X	X			El, Mi	Sc, Gr	High	42
Combe-Grenal	16	France	Mou	Layer 11-26	X	X		X	El, Mi	Sc, Gr	High	(40, 42)
Caminade-Est	17	France	Mou	2 and 3	X					Sc	High	(46, 133)
Pech de l’Azé I	18	France	Mou	Levels 4-7	X	X			El, Mi	Gr, Sc, Sm	High	(43, 46)
Pech de l'Azé IV	18	France	Mou	J3a, 6A, F1, F3, F4, I1, I2 , J1	X	X			El, Mi	Gr, Sc, Sm	High	46
La Chapelle-aux-Saints	19	France	Mou	Unknown		X				Indet	Med	46
Ormesson	20	France	Mou	Pits 3 and 7		X	X		Mi	Sc	High	41
Grotte du Renne	21	France	Mou	XI-XVI	X	X	X		Mi	Sm, Heat	Med	134
Grotte du Bison	21	France	Mou	E-J	X	X	X			Indet	Med	134
Grotte de l'Ermitage	22	France	Mou	Unknown	X	X				Indet	Med	46
Scladina	23	Belgium	Mou	Unit 1 a and Z-Inf	X				El, Mi	Sm	High	135
Maastricht-Belvédère	24	Netherlands	Mou	Site C		X			El, Mi	Pulv, Mix	High	3
Achenheim	25	France	Mou	At. loess with large shells		X				Gr	Low	36
Fumane Cave	26	Italy	Mou	A9		X			El, Mi	Pulv, Paint	High	49
Beçov I	27	Czech Republ	Mou	Layer A-III-6		X	X	X	El, Mi	Kn ?	Low	(37, 38, 39, 122)
Raj Cave	28	Poland	Mou	Upper 6		X				Gr	Low	136
Cioarei- Borosteni Cave	29	Romania	Mou	X-XIII		X			Mi	Pulv, Mix	High	(137, 138)
Molodova 1	30	Ukraine	Mou	4		X				Indet	Low	(139, 140)
Ilskaya 1	31	Russia	Mou	LW		X				Indet	Low	123
Shlyakh	32	Russia	Mou	8		?				Indet	Med	123
Volgograd/Stalingradskay	33	Russia	Mou	Unknown		X				Indet	Low	123
Station des rochettes	*	France	Mou	6		X				Sc	Med	141
Mukhovets	MU	Ukraine	Mou	B8-B2		X				Indet	Low	142
Prolom II	PRI	Ukraine	Mou	I-IV		X	X			Sm, Kn	High	(71, 118)
Zaskalanaya V	ZSK	Ukraine	Mou	II, IV, V, Trench		X	X			Eng, Sc, Sm, Gr, Kn	High	(68, 70)
Zaskalanaya VI	ZSK	Ukraine	Mou	I-V		X	X			Sc, Sm, Kn	High	(70, 73)

Table S1.

Acheulean and Mousterian sites with claimed and demonstrated evidence of coloring materials exploitation. *: Not presented in Fig. 1; At.: atypical; B: black; R: red; Y: yellow; W: white; El: elemental; Mi: mineralogical; Sm: smoothing; Sc: scraping; Gr: grinding; Kn: Knapping; Eng: Engraving; Heat: Heating; Pulv: pulverizing; Mix: mixing; Paint: painting; a: Transport smoothing; b: Probable engraving; Conf.: Index of confidence in the ochre modifications.

Site	Layer	Material	Laboratory no	14C Age	Calibrated age IntCAl20 (95.4)	Reference
ZSKV	I	B	Ki-10891	28,850 ± 400	31,220 ± 593	109
	I	B	Ki-10744	30,080 ± 350	32,647 ± 336	109
	II	B	Ki-10743	31,600 ± 350	33,995 ± 357	109
	II	B	OxA-35786	41,600 ± 1,400	42,929 ± 1,398	98
	II	C	OxA-35253	37,350 ± 500	39,978 ± 281	98
	III	C	OxA-35602	32,000 ± 800	34,769 ± 1,043	98
	III	C	OxA-35526	42,700 ± 1,100	43,664 ± 1,066	98
	III	B	Ki-10603	39,200 ± 520	40,872 ± 339	109
	IV	BB	GrA-13916	>46,000		101
	IV	B	Ki-10603	>47,000		109
ZSKVI	I	B	Ki-10892	22,500 ± 450	24,828 ± 436	109
	I	B	Ki-10605	22,800 ± 400	25,089 ± 413	109
	I	B	Ki-10606	24,400 ± 480	26,690 ± 524	109
	I	B	Ki-13373	25,700 ± 160	28,035 ± 175	70
	I	B	Ki-13375	25,200 ± 160	27,565 ± 213	70
	I	B	Ki-13376	24,600 ± 170	26,914 ± 176	70
	II	B	OxA-4131	30,110 ± 630	32,785 ± 374	(107, 143)
	II	B	Ki-10607	30,220 ± 400	32,785 ± 374	109
	II	B	Ki-10893	30,700 ± 450	33,191 ± 437	109
	II	B	Ki-10608	31,100 ± 490	33,556 ± 494	109
	III	B	OxA-4772	35,250 ± 900	38,386 ± 865	(109, 143)
	III	B	Ki-10894	36,400 ± 450	39,455 ± 342	109
	III	B	Ki-10609	38,200 ± 410	40,402 ± 164	109
	IIIa	B	OxA-4132	30,760 ± 690	33,336 ± 682	(107, 143)
	IIIa	B	OxA-4773	39,100 ± 1,500	41,436 ± 1,238	(107, 143)
	IIIa	B	Ki-10610	39,400 ± 480	40,970 ± 351	109
	IV	B	KI-10611	>47,000		109
PRMII	I	B	Ki-10746	22,900 ± 300	25,211 ± 332	109
	I	BB	GrA-5445	22,650 ± 100	30,137 ± 281	101
	I	B	Ki-10895	22,800 ± 600	25,131 ± 606	109
	I	B	Ki-10745	24,550 ± 300	26,809 ± 363	109
	II	B	Ki-10617	28,100 ± 300	30,315 ± 465	109
	III	B	Ki-10611	41,600 ± 800	42,527 ± 698	109

Table S2.

Radiocarbon ages for Zaskalnaya V, Zaskalnaya VI and Prolom II.

Site	Sample ID	Layer	Industry	Humans (MNI)
ZSKV	ZSKV-09	II	Mi	
	ZSKV-05	V	Mi	1 child
	ZSKV-07	V	Mi	
	ZSKV-74	V	Mi	
	ZSKV-10D	V	Mi	
	ZSKV-08	VI	Mi	
	ZSKV-06	Trench	Mi	1
ZSKVI	ZSKVI-01	II	Mi	
	ZSKVI-04	II	Mi	3
	ZSKVI-03	II	Mi	
	ZSKVI-02	III/IIIa	Mi	
	ZSKVI-38	IIIa	Mi	5 to 7
PRMII	PRM-9e	II	Mi	1
	PRM-11e	II	Mi	
MUH	MUH12	680-700	Mou	
	MUH13	700-720	Mou	
ZSKV: Zaskalnaya V; ZSKVI: Zaskalnaya VI; PRMII: Prolom II;				
MUH: Mukhovets; Mi: Micoquian				

Table S3.

Archaeological context of the studied coloring materials.

	Description				Semi-quantitative EDX analyses **				Interpretation
Reference	Analysed area	Grain morphology	BSE * contrast	Grain size (µm)	>10%	3-10%	3-1%	<1%	
ZSK-V-6	Zone 1 Zoom 3 Point 1	Regular	Light grey	2.8 x 2.8	**Fe**	(Si)	(Al, Ca)	Mn, (Mg, K, P, Ti, S)	Iron oxide
ZSK-V-6	Zone 1 Zoom 3 Point 2	Agglomerate of sub-micrometric grains	Grey	-	**Fe**	Si	Ca, Al	Mn, Mg, (P), K, (S), Ti	Clay minerals + iron oxides
ZSK-V-6	Zone 1 Zoom 3 Point 3	Platy	Dark grey	4 x 2.3	(Fe), Si, Al	K	Ca	Mg, Ti, (Mn, P, S)	K-rich mica
ZSK-V-6	Zone 2 Zoom 6 Point 1	Amorphous	Light grey	-	**Ca**	(Fe, Si)	S, (Al)	(P, K, Mg, Na)	Ca-rich sulphate
ZSK-V-6	Zone 2 Zoom 6 Point 2	Amorphous	Light grey	-	**Ca**		(Fe, Si), S	(P, Al, Mg, Na, K)	Ca-rich sulphate
ZSK-V-6	Zone 3 Zoom 8 Point 1	Platy	Dark grey	7.6 x 9.7	**(Fe)**	(Si)	(Ca, Al)	(P, Mn, K, Mg, Ti, S, Na)	Carbon ?
ZSK-V-6	Zone 3 Zoom 8 Point 2	Regular	Light grey	9.8 x 6.6	(Fe), Si	Al, K	Ca	Mg, (P)	K-rich feldspar
ZSK-V-6	Zone 3 Zoom 8 Point 3	Agglomerate of sub-micrometric grains	Grey	-	**Fe**	Si, Al	Ca	Mg, Mn, K, (P), Ti, (S), Na	Clay minerals + iron oxides
ZSK-V-7	Zone 1 Zoom 5 Point 1	Agglomerate of sub-micrometric grains	Grey	-	**Fe**	(Ca), Si	(P), Al	K, Mg	Clay minerals + iron oxides
ZSK-V-7	Zone 1 Zoom 5 Point 2	Regular	Light grey	1.2 x 0.8	**Fe**	(Si)	(Ca, P)	(Al, Na, Mg)	Iron oxide
ZSK-V-7	Zone 1 Zoom 5 Point 3	Sub-circular	Grey	1.4 x 0.8	**Fe**	(Si, Ca)	(Al, P)	(Mg, K, Na, S)	Iron oxide
ZSK-V-5	Zone 1 Zoom3 Point 1	Platy	Grey	17 x 11.7	Si, Fe, Al		Ca	Mg, K, (P)	Mica
ZSK-V-5	Zone 1 Zoom3 Point 2	Irregular	Light grey	4.8 x 2.7	**(Ca),** Fe	(Si)	(Al)	(Mg, P, K, Na)	Iron oxide
ZSK-V-5	Zone 1 Zoom3 Point 3	Irregular	Grey	8 x 8	**Fe**	(Si, Al)	(K, Ca, Mg)	(P, Na)	Iron oxide
ZSK-VI-1	Zone 1 Zoom3 Point 1	Platy	Black	23 x 22	(Si, Fe)		(Al)	(Ca, Mg, K, Na)	Carbon ?
ZSK-VI-1	Zone 1 Zoom3 Point 2	Irregular	Grey	22 x 11	**(Fe,** Si)	(Al)	(Ca)	(Mg, K, Mn, P, Na)	Silicate
ZSK-VI-1	Zone 1 Zoom3 Point 3	Irregular	Light grey	1.4 x. 1.1	Fe, (Si, Al)		(K, Ca, Mg)	(P, Na)	Iron oxide

(*): White, Grey, and Black refer to the contrast observed on backscattered electron (BSE) images. Weight percentatges including O, normalized to 100%.

(**): Elements in brackets play no role in the mineralogical composition of the analysed items. Elements in bold are present in a proportion equal or higher than 40%.

Table S4.

Results of semi-quantitative SEM-EDS analysis of ochre pieces from ZSK V and ZSK VI.

Standard		SiO_2 (%)	K_2O (%)	CaO (%)	TiO_2 (%)	MnO (%)	Fe_2O_3 (%)	V (ppm)	Cr (ppm)	Ni (ppm)	Cu (ppm)	Zn (ppm)	Ga (ppm)	As (ppm)	Rb (ppm)	Sr (ppm)	Y (ppm)	Zr (ppm)	Ba (ppm)
DR-N	Measures	**47.94 ± 1.01**	1.49 ± 0.01	6.53 ± 0.07	1.06 ± 0.02	0.23*	**7.86 ± 0.04**	191 ± 20	<LOD	<LOD	<LOD	**44 ± 24**	20 ± 2	**15 ± 2**	63 ± 2	367 ± 9	24 ± 11	159 ± 32	**225 ± 57**
	Certified	**52.85 ± 0.73**	1.70 ± 0.10	7.05 ± 0.24	1.09 ± 0.08	0.22 ± 0.02	**9.7 ± 0.29**	220 ± 34	40 ± 10	15 ± 11	50 ± 7	**145 ± 16**	22 ± 5	**3 ±1**	73 ± 8	400 ± 50	26 ± 7	125 ± 25	**385 ± 12**
SARM69	Measures	68.66 ± 0.96	1.85 ± 0.03	2.68 ± 0.06	0.82 ± 0.02	0.17*	6.31 ± 0.20	170 ± 22	**123 ± 13**	**15 ± 1**	**129 ± 150**	<LOD	<LOD	<LOD	68 ± 3	**56 ± 2**	27 ± 5	**177 ± 20**	**240 ± 29**
	Certified	64.16 ± 3.37	1.72 ± 0.41	2.92 ± 1.92	0.84 ± 0.14	0.13 ± 0.03	7.40 ± 1.58	198 ± 47	**330 ± 118**	**60 ± 14**	**48 ± 15**	66 ± 11	**	**	52 ± 10	**107 ± 17**	31 ± 10	**268 ± 29**	**573 ± 125**
Mica-Mg	Measures	34.35 ± 1.56	10.35 ± 0.08	0.04 ± 0.01	1.46 ± 0.02	0.28*	**7.06 ± 0.04**	**202 ± 8**	**8 ± 5**	**29 ± 1**	<LOD	**472 ± 32**	22 ± 1	9***	**1044±5**	31±2	<LOD	**130 ± 24**	**1774 ± 95**
	Certified	38.30 ± 0.36	10.00 ± 0.29	0.08 ± 0.04	1.63 ± 0.10	0.26 ± 0.04	**9.46 ± 0.30**	**90 ± 63**	**100 ± 66**	**110 ± 18**	4 ± 10	**290 ± 35**	21 ± 7	**	**1300±114**	27±7	**	**16 ± 9**	**4000 ± 552**
BX-N	Measures	10.99± 3.65	<LOD	0.12 ± 0.01	2.77 ± 0.03	0.03*	23.29 ± 0.20	375 ± 8	**104 ± 3**	157 ± 4	**133 ± 7**	**154 ± 2**	50 ± 1	**186 ± 2**	<LOD	102 ± 5	140 ± 2	410 ± 5	<LOD
	Certified	7.40 ± 0.50	0.05 ± 0.03	0.17 ± 0.14	2.37 ± 0.23	0.05 ± 0.02	23.17 ± 0.84	350 ± 77	**280 ± 75**	180 ± 37	**18 ± 4**	**80 ± 39**	67 ± 19	**115 ± 9**	4 ± 11	110 ± 19	114 ± 40	550 ± 89	30 ± 25
Mica-Fe	Measures	**41.01 ± 0.24**	8.31 ± 0.11	0.29 ± 0.01	2.11 ± 0.02	0.33*	27.03 ± 0.35	79 ± 19	<LOD	<LOD	<LOD	1257 ± 20	99 ± 15	**21 ± 2**	1995 ± 22	8***	35 ± 4	986 ± 18	**368 ± 47**
	Certified	**34.40 ± 0.68**	8.75 ± 0.35	0.43 ± 0.11	2.50 ± 0.20	0.35 ± 0.04	25.65 ± 0.77	135 ± 64	90 ± 84	35 ± 19	5 ± 12	1300 ± 220	95 ± 26	**3 ± 5**	2200 ± 269	5 ± 9	48 ± 40	800 ± 234	**150 ± 90**

Table S5.

Results of the pXRF elemental analysis of the pXRF standards. Mean and standard deviation elemental concentrations in standard samples. *SD<0.01 %, ** No certified value, *** SD<1 ppm., <LOD values below limit of detection, measures and certified values in bold differ one to another.

ID	Sample	Site	Lithology	Color	Modification index	Abrasion index	Fragm. index	Year	Fe2O3	SiO2	CaO	MnO
XRF-ZSK-VI-01.01	VI-01	ZSK-VI	1a	Red	4a	2a	2	2015	1,580012113	-0,731822996	-1,66619826	-2,102890858
XRF-ZSK-VI-01.02	VI-01	ZSK-VI	1a	Red	4a	2a	2	2015	1,4363217	-0,560681763	-1,419288361	-2,056110458
XRF-ZSK-VI-01.03	VI-01	ZSK-VI	1a	Red	4a	2a	2	2015	1,32756326	-0,509337367	-1,441072535	-2,048809659
XRF-ZSK-VI-01.04	VI-01	ZSK-VI	1a	Red	4a	2a	2	2015	1,660580912	-0,656689746	-1,514452877	-2,183459658
XRF-ZSK-VI-01.05	VI-01	ZSK-VI	1a	Red	4a	2a	2	2015	1,671913012	-0,671913012	-1,31207753	-2,103711288
XRF-ZSK-VI-01.06	VI-01	ZSK-VI	1a	Red	4a	2a	2	2015	1,742410881	-0,77815125	-1,472897936	-2,18610838
XRF-ZSK-VI-01.07	VI-01	ZSK-VI	1a	Red	4a	2a	2	2015	1,730701544	-0,741696929	-1,368973708	-2,1866335
XRF-ZSK-VI-02.01	VI-02	ZSK-VI	1a	Red	0	0	0	2015	1,666892211	-0,617286598	-0,987464314	-2,098690487
XRF-ZSK-VI-02.02	VI-02	ZSK-VI	1a	Red	0	0	0	2015	1,702688968	-0,751351449	-1,092028805	-2,100628977
XRF-ZSK-VI-02.03	VI-02	ZSK-VI	1a	Red	0	0	0	2015	1,61898892	-0,483856269	-0,82172138	-1,956231089
XRF-ZSK-VI-02.04	VI-02	ZSK-VI	1a	Red	0	0	0	2015	1,61898892	-0,496773042	-0,824500874	-1,956231089
XRF-ZSK-VI-02.05	VI-02	ZSK-VI	1a	Red	0	0	0	2015	1,619615006	-0,503339418	-0,827223316	-1,956857174
XRF-ZSK-VI-02.06	VI-02	ZSK-VI	1a	Red	0	0	0	2015	1,656864492	-0,664753004	-0,537278717	-2,165502798
XRF-ZSK-VI-02.07	VI-02	ZSK-VI	1a	Red	0	0	0	2015	1,658393026	-0,696023691	-0,546794501	-2,167031332
XRF-ZSK-VI-02.08	VI-02	ZSK-VI	1a	Red	0	0	0	2015	1,668199484	-0,646596768	-1,172655147	-2,055415627
XRF-ZSK-VI-03.01	VI-03	ZSK-VI	4	Dark Red	1	0	1	2015	1,796227314	-0,774211574	-1,142050772	-2,434499478
XRF-ZSK-VI-03.02	VI-03	ZSK-VI	4	Dark Red	1	0	1	2015	1,793371249	-0,754353927	-1,137273047	-2,431643413
XRF-ZSK-VI-03.03	VI-03	ZSK-VI	4	Dark Red	1	0	1	2015	1,793371249	-0,760349804	-1,135359852	-2,431643413
XRF-ZSK-VI-03.04	VI-03	ZSK-VI	4	Dark Red	1	0	1	2015	1,830203599	-0,903861152	-1,464715614	-2,487780918
XRF-ZSK-VI-03.05	VI-03	ZSK-VI	4	Dark Red	1	0	1	2015	1,768786047	-0,724638426	-1,304893058	-2,467756051
XRF-ZSK-VI-03.06	VI-03	ZSK-VI	4	Dark Red	1	0	1	2015	1,7513561	-0,815848834	-1,68689811	-2,450326104
XRF-ZSK-VI-04.01	VI-04	ZSK-VI	2	Red	0	0	0	2015	0,850646235	-0,328202002	0,733005873	-2,005548195
XRF-ZSK-VI-04.02	VI-04	ZSK-VI	2	Red	0	0	0	2015	0,707570176	-0,159795471	0,871298153	-2,40654018
XRF-ZSK-VI-04.03	VI-04	ZSK-VI	2	Red	0	0	0	2015	0,709269961	-0,156601745	0,849798373	-2,232148706
XRF-ZSK-VI-04.04	VI-04	ZSK-VI	2	Red	0	0	0	2015	0,808210973	-0,119791151	0,741772638	-2,206150982
XRF-ZSK-VI-04.05	VI-04	ZSK-VI	2	Red	0	0	0	2015	0,807535028	-0,272240908	0,806412449	-2,108565024
XRF-ZSK-VI-04.06	VI-04	ZSK-VI	2	Red	0	0	0	2015	0,808210973	-0,272916853	0,807739079	-2,109240969
XRF-ZSK-V-05.01	V-05	ZSK-V	1a	Red	5b	3b	2	2015	1,717670503	-0,615236797	-0,774175987	-2,173602459
XRF-ZSK-V-05.02	V-05	ZSK-V	1a	Red	5b	3b	2	2015	1,736476182	-0,679571331	-0,783683739	-2,259354927
XRF-ZSK-V-05.03	V-05	ZSK-V	1a	Red	5b	3b	2	2015	1,747567163	-0,649269626	-0,825880687	-2,332593815
XRF-ZSK-V-05.04	V-05	ZSK-V	1a	Red	5b	3b	2	2015	1,805500858	-0,817834593	-1,107400313	-3,106530854
XRF-ZSK-V-05.05	V-05	ZSK-V	1a	Red	5b	3b	2	2015	1,822625679	-0,880121573	-1,349869229	-3,521595683
XRF-ZSK-V-06.01	V-06	ZSK-V	1b	Yellow	6	3	3	2015	1,725339816	-0,721018442	-0,870426794	-2,053241958
XRF-ZSK-V-06.02	V-06	ZSK-V	1b	Yellow	6	3	3	2015	1,744605875	-0,73899643	-0,965009384	-2,028602532
XRF-ZSK-V-06.03	V-06	ZSK-V	1b	Yellow	6	3	3	2015	1,734239604	-0,730779072	-1,108927153	-2,0352696
XRF-ZSK-V-07.01	V-07	ZSK-V	4	Dark Red	3	2	1	2015	1,751663946	-0,751663946	-0,946843268	-3,751663946
XRF-ZSK-V-07.02	V-07	ZSK-V	4	Dark Red	3	2	1	2015	1,78490245	-0,874278045	-1,131689936	-3,78490245
XRF-ZSK-V-07.03	V-07	ZSK-V	4	Dark Red	3	2	1	2015	1,809088131	-0,929418926	-0,940443693	-3,809088131
XRF-ZSK-V-07.04	V-07	ZSK-V	4	Dark Red	3	2	1	2015	1,671913012	-0,493511671	-0,741983452	-3,671913012
XRF-ZSK-V-08.01	V-08	ZSK-V	1b	Yellow	4b	2b	2	2015	1,755874856	-0,65966027	-1,579783597	-2,278753601
XRF-ZSK-V-08.02	V-08	ZSK-V	1b	Yellow	4b	2b	2	2015	1,7084209	-0,597158386	-1,462908232	-2,246022902
XRF-ZSK-V-08.03	V-08	ZSK-V	1b	Yellow	4b	2b	2	2015	1,675595056	-0,486385567	-1,568385087	-2,198473802
XRF-ZSK-V-08.04	V-08	ZSK-V	1b	Yellow	4b	2b	2	2015	1,694956002	-0,53328859	-1,507435281	-2,203594308
XRF-ZSK-V-08.05	V-08	ZSK-V	1b	Yellow	4b	2b	2	2015	1,680969718	-0,501417927	-1,525633681	-2,189608025
XRF-ZSK-V-09.01	V-09	ZSK-V	1b	Yellow	0	0	0	2015	1,663040975	-0,419011386	-0,778245611	-1,469916377
XRF-ZSK-V-09.02	V-09	ZSK-V	1b	Yellow	0	0	0	2015	1,66829271	-0,447661691	-0,724798195	-1,574871025
XRF-ZSK-V-09.03	V-09	ZSK-V	1b	Yellow	0	0	0	2015	1,654272827	-0,397795621	-0,917876325	-1,616846329
XRF-ZSK-V-09.04	V-09	ZSK-V	1b	Yellow	0	0	0	2015	1,727622578	-0,651710816	-1,072484143	-2,037426498
XRF-ZSK-V-09.05	V-09	ZSK-V	1b	Yellow	0	0	0	2015	1,734879803	-0,613976985	-0,859239866	-2,010603933
XRF-ZSK-V-09.06	V-09	ZSK-V	1b	Yellow	0	0	0	2015	1,722304787	-0,600088909	-0,88534805	-2,059546955
XRF-ZSK-V-10D.01	V-10D	ZSK-V	1b	Yellow	3a	2a	1	2017	1,636888907	-0,609539299	-1,419404963	-1,896526217
XRF-ZSK-V-10D.02	V-10D	ZSK-V	1b	Yellow	3a	2a	1	2017	1,543819805	-0,771498098	-1,461034435	-1,744479256
XRF-ZSK-V-10D.03	V-10D	ZSK-V	1b	Yellow	3a	2a	1	2017	1,509202522	-0,538855646	-1,456124079	-1,716810833
XRF-ZSK-V-10D.04	V-10D	ZSK-V	1b	Yellow	3a	2a	1	2023	1,630732893	-0,461058459	-1,413248949	-1,931762888
XRF-ZSK-V-10D.05	V-10D	ZSK-V	1b	Yellow	3a	2a	1	2023	1,623352682	-0,465291888	-1,581959996	-2,010568825
XRF-ZSK-V-10D.06	V-10D	ZSK-V	1b	Yellow	3a	2a	1	2023	1,627263417	-0,46172234	-1,544478046	-2,004014126
XRF-ZSK-V-10D.07	V-10D	ZSK-V	1b	Yellow	3a	2a	1	2023	1,588383768	-0,478805221	-1,535305325	-1,986323777
XRF-ZSK-V-74.01	V-74	ZSK-V	1b	Yellow	1	0	1	2017	1,545801757	-0,629874545	-1,320492475	-1,966018161
XRF-ZSK-V-74.02	V-74	ZSK-V	1b	Yellow	1	0	1	2017	1,541079768	-0,613709405	-1,257778539	-1,961296171
XRF-ZSK-V-74.03	V-74	ZSK-V	1b	Yellow	1	0	1	2023	1,49762065	-0,731952095	-0,486050206	-1,884836793
XRF-ZSK-V-74.04	V-74	ZSK-V	1b	Yellow	1	0	1	2023	1,488832634	-0,853348888	-0,479381739	-1,886772643
XRF-ZSK-V-74.05	V-74	ZSK-V	1b	Yellow	1	0	1	2023	1,503109437	-0,714234321	-0,506160188	-1,879860146
XRF-ZSK-VI-38.01	VI-38	ZSK-VI	4	Black	1	0	1	2015	1,785044958	-1,427110111	-0,569407395	-2,671101606
XRF-ZSK-VI-38.02	VI-38	ZSK-VI	4	Black	1	0	1	2015	1,80106053	-1,448878012	-0,544342784	-2,759667845
XRF-ZSK-VI-38.03	VI-38	ZSK-VI	4	Black	1	0	1	2015	1,771954749	-1,439516289	-0,565938872	-2,692773503
XRF-ZSK-VI-38.04	VI-38	ZSK-VI	4	Black	1	0	1	2015	1,792601781	-1,456142047	-0,265616713	-2,889511794
XRF-ZSK-VI-38.05	VI-38	ZSK-VI	4	Black	1	0	1	2015	1,737272177	-1,408892573	-0,270552805	-3,135212185
XRF-PRM-II-9e.01	II-9e	PRMII	3	Black	1	0	1	2023	1,504470862	-0,315542379	-0,792663633	-1,495870691
XRF-PRM-II-9e.02	II-9e	PRMII	3	Black	1	0	1	2023	1,531989551	-0,352725087	-0,794002225	-1,490596866
XRF-PRM-II-9e.03	II-9e	PRMII	3	Black	1	0	1	2023	1,529558673	-0,3118116	-0,821988497	-1,48034065
XRF-PRM-II-9e.04	II-9e	PRMII	3	Black	1	0	1	2023	1,504742636	-0,311061607	-0,79717246	-1,467316138
XRF-PRM-II-9e.05	II-9e	PRMII	3	Black	1	0	1	2023	1,5774918	-0,384645685	-0,822379533	-1,395648212
XRF-PRM-II-9e.06	II-9e	PRMII	3	Black	1	0	1	2023	1,558588583	-0,373613392	-0,835954661	-1,431483785
XRF-PRM-II-11e.01	II-11e	PRMII	3	Black	1	1	0	2023	1,3232521	-0,243709093	-0,173417403	-1,183373014
XRF-PRM-II-11e.02	II-11e	PRMII	3	Black	1	1	0	2023	1,32776749	-0,227396945	-0,162226413	-1,181639454
XRF-PRM-II-11e.03	II-11e	PRMII	3	Black	1	1	0	2023	1,295127085	-0,138880895	-0,127809751	-1,133759083
XRF-PRM-II-11e.04	II-11e	PRMII	3	Black	1	1	0	2023	1,266701967	-0,119644296	-0,131569316	-1,123687167
XRF-PRM-II-11e.05	II-11e	PRMII	3	Black	1	1	0	2023	1,266701967	-0,104735351	-0,123999721	-1,120573931
XRF-MUH-12.01	12	MUH	5	Red	2	2	0	2015	1,133538908	0,312998259	0,110243008	-1,929418926
XRF-MUH-12.02	12	MUH	5	Red	2	2	0	2015	1,260071388	0,125892183	0,061527042	-1,981317787
XRF-MUH-12.03	12	MUH	5	Red	2	2	0	2015	1,266231697	0,031091018	0,172468836	-1,886020455
XRF-MUH-12.04	12	MUH	5	Red	2	2	0	2015	1,275311355	0,012937871	0,164652581	-1,913583519
XRF-MUH-12.05	12	MUH	5	Red	2	2	0	2015	1,276691529	0,023903955	0,153383527	-1,914963693
XRF-MUH-12.06	12	MUH	5	Red	2	2	0	2015	1,140193679	0,177033671	0,098604884	-1,994065643
XRF-MUH-12.07	12	MUH	5	Red	2	2	0	2015	1,264109156	0,086913696	0,11501499	-1,963079161
XRF-MUH-13.01	13	MUH	6	Red	0	0	0	2015	0,696356389	-0,202201795	0,915154498	-1,128154665
XRF-MUH-13.02	13	MUH	6	Red	0	0	0	2015	0,673020907	-0,187299481	0,950124968	-1,350801612
XRF-MUH-13.03	13	MUH	6	Red	0	0	0	2015	0,813580989	-0,3379098	0,828388609	-1,269512944
XRF-MUH-13.04	13	MUH	6	Red	0	0	0	2015	0,8162413	-0,319311652	0,82114835	-1,272173256
XRF-MUH-13.05	13	MUH	6	Red	0	0	0	2015	0,746634199	-0,240129167	0,70191754	-1,241484221
XRF-MUH-13.06	13	MUH	6	Red	0	0	0	2015	0,887054378	-0,435267943	0,712391998	-1,506843136
XRF-MUH-13.07	13	MUH	6	Red	0	0	0	2015	0,825426118	-0,297796217	0,826433151	-1,427486109
XRF-Geol-Ref01.01	Ref01	Geological	Geological	Geological	Geological	Geological	Geological	2015	1,811038509	-1,224451204	-2,018646819	-1,930224916
XRF-Geol-Ref01.02	Ref01	Geological	Geological	Geological	Geological	Geological	Geological	2015	1,721975402	-0,710404958	-1,704942062	-2,023005397
XRF-Geol-Ref01.03	Ref01	Geological	Geological	Geological	Geological	Geological	Geological	2015	1,716670976	-0,726776412	-1,83018025	-2,035429738
XRF-Geol-Ref01.04	Ref01	Geological	Geological	Geological	Geological	Geological	Geological	2015	1,722057771	-0,729504253	-1,782538519	-2,023087767
XRF-Geol-Ref01.05	Ref01	Geological	Geological	Geological	Geological	Geological	Geological	2023	1,739097446	-0,991685638	-1,852606721	-1,932917472
XRF-Geol-Ref01.06	Ref01	Geological	Geological	Geological	Geological	Geological	Geological	2023	1,739018246	-1,044413047	-1,863956982	-1,939677696
XRF-Geol-Ref01.07	Ref01	Geological	Geological	Geological	Geological	Geological	Geological	2023	1,761777538	-0,993879921	-1,847963685	-1,942233602
XRF-Geol-Ref01.08	Ref01	Geological	Geological	Geological	Geological	Geological	Geological	2023	1,728516105	-1,023508145	-1,847702512	-1,94318627
XRF-Geol-Ref02.01	Ref02	Geological	Geological	Geological	Geological	Geological	Geological	2017	1,703549298	-0,963976954	-1,925398048	-1,858451258
XRF-Geol-Ref02.02	Ref02	Geological	Geological	Geological	Geological	Geological	Geological	2017	1,708845638	-0,978871352	-1,930694388	-1,863747598
XRF-Geol-Ref02.03	Ref02	Geological	Geological	Geological	Geological	Geological	Geological	2017	1,702775078	-0,998624561	-1,924623828	-1,870266165
XRF-Geol-Ref02.04	Ref02	Geological	Geological	Geological	Geological	Geological	Geological	2023	1,703635238	-0,910543637	-1,800545251	-1,932783226
XRF-Geol-Ref02.05	Ref02	Geological	Geological	Geological	Geological	Geological	Geological	2023	1,633165354	-0,763933634	-1,746674629	-1,91716201
XRF-Geol-Ref02.06	Ref02	Geological	Geological	Geological	Geological	Geological	Geological	2023	1,632558515	-0,76745454	-1,740463912	-1,93358851
XRF-Geol-Ref02.07	Ref02	Geological	Geological	Geological	Geological	Geological	Geological	2023	1,629001619	-0,9180385	-1,748188027	-1,930031615
XRF-Geol-Ref03.01	Ref03	Geological	Geological	Geological	Geological	Geological	Geological	2017	1,626134979	-0,611194629	-2,013351122	-1,800060176
XRF-Geol-Ref03.02	Ref03	Geological	Geological	Geological	Geological	Geological	Geological	2017	1,639884742	-0,629160877	-2,016635452	-1,807375829
XRF-Geol-Ref03.03	Ref03	Geological	Geological	Geological	Geological	Geological	Geological	2023	1,631646663	-0,747417893	-1,792797572	-1,839254973
XRF-Geol-Ref03.04	Ref03	Geological	Geological	Geological	Geological	Geological	Geological	2023	1,631646663	-0,695635867	-1,774314167	-1,825466689
XRF-Geol-Ref03.05	Ref03	Geological	Geological	Geological	Geological	Geological	Geological	2023	1,648457594	-0,739972575	-1,767644002	-1,835544238
XRF-Geol-Ref03.06	Ref03	Geological	Geological	Geological	Geological	Geological	Geological	2023	1,644044493	-0,748621947	-1,774812773	-1,837864519
XRF-Geol-Ref03.07	Ref03	Geological	Geological	Geological	Geological	Geological	Geological	2023	1,640382045	-0,764164204	-1,795284005	-1,841041495
XRF-Geol-Ref03.08	Ref03	Geological	Geological	Geological	Geological	Geological	Geological	2023	1,629409599	-0,665149969	-2,097930682	-1,61237626

Table S6.

Results of the pXRF elemental analysis of archaeological and geological ochre samples. The values presented in this table are those used to generate the plots, having undergone log transformation and normalization to iron (Fe). Iron itself is presented in logarithmic form without additional normalization. These transformations are described in the methods section.

REFERENCES AND NOTES

1. S. McBrearty, A. S. Brooks, The revolution that wasn't: A new interpretation of the origin of modern human behavior. *J. Hum. Evol.* **39**, 453–563 (2000).

2. F. d'Errico, C. B. Stringer, Evolution, revolution or saltation scenario for the emergence of modern cultures? *Philos. Trans. R. Soc. B Biol. Sci.* **366**, 1060–1069 (2011).

3. W. Roebroeks, M. J. Sier, T. K. Nielsen, D. De Loecker, J. M. Parés, C. E. S. Arps, H. J. Mücher, Use of red ochre by early Neandertals. *Proc. Natl. Acad. Sci. U.S.A.* **109**, 1889–1894 (2012).

4. S. Thavapalan, D. A. Warburton, *The Value of Colours. Material and Economic Aspects in the Ancient World* (Humboldt-Universität zu Berlin, 2019); https://edoc.hu-berlin.de/handle/18452/21360.

5. R. Dapschauskas, M. B. Göden, C. Sommer, A. W. Kandel, The emergence of habitual ochre use in Africa and its significance for the development of ritual behavior during the Middle Stone Age. *J. World Prehistory* **35**, 233–319 (2022).

6. P. Kay, B. Berlin, W. Merrifield, Biocultural implications of systems of color naming. *J. Linguist. Anthropol.* **1**, 12–25 (1991).

7. J. Maule, A. E. Skelton, A. Franklin, The development of color perception and cognition. *Annu. Rev. Psychol.* **74**, 87–111 (2023).

8. P. Kay, R. S. Cook, "World color survey," in *Encyclopedia of Color Science and Technology* (Springer, 2023), pp. 1601–1607. https://link.springer.com/rwe/10.1007/978-3-030-89862-5_113.

9. K. Nassau, *Color for Science, Art and Technology* (Elsevier, 1997).

10. E. Telles, *Pigmentocracies: Ethnicity, Race, and Color in Latin America* (UNC Press Books, 2014).

11. R. MacLaury, G. Paramei, D. Dedrick, Eds., *Anthropology of Color: Interdisciplinary Multilevel Modeling* (John Benjamins Publishing Company, 2007); https://library.oapen.org/handle/20.500.12657/30730.

12. T. W. Deacon, *The Symbolic Species: The Co-Evolution of Language and the Brain* (W.W. Norton & Company, 1997).

13. E. C. Velliky, B. L. MacDonald, M. Porr, N. J. Conard, First large-scale provenance study of pigments reveals new complex behavioural patterns during the Upper Palaeolithic of south-western Germany. *Archaeometry* **63**, 173–193 (2021).

14. R. S. Popelka-Filcoff, A. M. Zipkin, The archaeometry of ochre *sensu lato*: A review. *J. Archaeol. Sci.* **137**, 105530 (2022).

15. L. Wadley, W. Bronwynne, M. Lombard, Ochre in hafting in Middle Stone Age southern Africa: A practical role. *Antiquity* **78**, 661–675 (2004).

16. R. F. Rifkin, Assessing the efficacy of red ochre as a prehistoric hide tanning ingredient. *J. Afr. Archaeol.* **9**, 131–158 (2011).

17. S. Soriano, P. Villa, L. Wadley, Ochre for the toolmaker: Shaping the Still Bay points at Sibudu (KwaZulu-Natal, South Africa). *J. Afr. Archaeol.* **7**, 41–54 (2009).

18. E. Hovers, S. Ilani, O. Bar-Yosef, B. Vandermeersch, An early case of color symbolism: Ochre use by modern humans in Qafzeh Cave. *Curr. Anthropol.* **44**, 491–522 (2003).

19. F. d'Errico, H. Salomon, C. Vignaud, C. Stringer, Pigments from the Middle Palaeolithic levels of Es-Skhul (Mount Carmel, Israel). *J. Archaeol. Sci.* **37**, 3099–3110 (2010).

20. J. Watts, O. Sheehan, Q. D. Atkinson, J. Bulbulia, R. D. Gray, Ritual human sacrifice promoted and sustained the evolution of stratified societies. *Nature* **532**, 228–231 (2016).

21. H. Salomon, C. Vignaud, Y. Coquinot, L. Beck, C. Stringer, D. Strivay, F. d'Errico, Selection and heating of colouring materials in the Mousterian level of Es-Skhul (c. 100 000 years BP, Mount Carmel, Israel). *Archaeometry* **54**, 698–722 (2012).

22. L. Dayet, P.-J. Texier, F. Daniel, G. Porraz, Ochre resources from the Middle Stone Age sequence of Diepkloof Rock Shelter, Western Cape, South Africa. *J. Archaeol. Sci.* **40**, 3492–3505 (2013).

23. G. Mauran, B. Caron, F. Détroit, A. Nankela, J.-J. Bahain, D. Pleurdeau, M. Lebon, Data pretreatment and multivariate analyses for ochre sourcing: Application to Leopard Cave (Erongo, Namibia). *J. Archaeol. Sci. Rep.* **35**, 102757 (2021).

24. D. I. Godfrey-Smith, S. Ilani, Past thermal history of goethite and hematite fragments from Qafzeh Cave deduced from thermal activation characteristics of the 110°C TL peak of enclosed quartz grains. *ArchéoSciences Rev. Archéom.* **28**, 185–190 (2004).

25. L. Wadley, Post-depositional heating may cause over-representation of red-coloured ochre in stone age sites. *South Afr. Archaeol. Bull.* **64**, 166–171 (2009).

26. F. d'Errico, M. Vanhaeren, N. Barton, A. Bouzouggar, H. Mienis, D. Richter, J.-J. Hublin, S. P. McPherron, P. Lozouet, Additional evidence on the use of personal ornaments in the Middle Paleolithic of North Africa. *Proc. Natl. Acad. Sci. U.S.A.* **106**, 16051–16056 (2009).

27. F. d'Errico, K. L. van Niekerk, L. Geis, C. S. Henshilwood, New Blombos Cave evidence supports a multistep evolutionary scenario for the culturalization of the human body. *J. Hum. Evol.* **184**, 103438 (2023).

28. T. E. Steele, E. Alvarez Fernández, E. Hallet-Desguez, A review of shells as personal ornamentation during the African Middle Stone Age. *PaleoAnthropology* **2019**, 24–51 (2019).

29. G. Mauran, M. Lebon, O. Lapauze, A. Nankela, F. Détroit, J. Lesur, J.-J. Bahain, D. Pleurdeau, Archaeological ochres of the rock art site of Leopard Cave (Erongo, Namibia): Looking for Later Stone Age sociocultural behaviors. *Afr. Archaeol. Rev.* **37**, 527–550 (2020).

30. A. Mackay, A. Welz, Engraved ochre from a Middle Stone Age context at Klein Kliphuis in the Western Cape of South Africa. *J. Archaeol. Sci.* **35**, 1521–1532 (2008).

31. C. S. Henshilwood, F. d'Errico, I. Watts, Engraved ochres from the Middle Stone Age levels at Blombos Cave, South Africa. *J. Hum. Evol.* **57**, 27–47 (2009).

32. C. S. Henshilwood, F. d'Errico, K. L. van Niekerk, L. Dayet, A. Queffelec, L. Pollarolo, An abstract drawing from the 73,000-year-old levels at Blombos Cave, South Africa. *Nature* **562**, 115–118 (2018).

33. D. E. Rosso, F. d'Errico, A. Queffelec, Patterns of change and continuity in ochre use during the late Middle Stone Age of the Horn of Africa: The Porc-Epic Cave record. *PLOS ONE* **12**, e0177298 (2017).

34. H. de Lumley, F. Audubert, S. Khatib, C. Perrenoud, B. Roussel, T. Saos, A. Szelewa, "Les crayons d'ocre du site acheuléen de Terra Amata," in *Terra Amata, Nice, Alpes-Maritimes, France Tome V,* Dir. H. de Lumley, C. Editions, Eds. (Histoire naturelle de l'Homme préhistorique, 2016), chap. 44, vol. 5, pp. 233–277. https://hal.science/hal-02999462. [The ochre crayons from the Acheulean site of Terra Amata].

35. F. C. Howell, Observations on the earlier phases of the European Lower Paleolithic. *Am. Anthropol.* **68**, 88–201 (1966).

36. A. Thévenin, "Les civilisations du Paléolithique moyen en Alsace," in *La préhistoire française. Les civilisations paléolithiques et mésolithiques de la France*, H. de Lumley, Ed. (CNRS, 1976), vol. 1, pp. 1139–1141. [Middle Paleolithic civilizations in Alsace].

37. A. Marshack, On Paleolithic ochre and the early uses of color and symbol. *Curr. Anthropol.* **22**, 188–191 (1981).

38. A. Šajnerová-Dušková, J. Fridrich, I. Fridrichová-Sýkorová, "Pitted and grinding stones from Middle Palaeolithic settlements in Bohemia: A functional study," in *Proceedings of the XV. Congress of the U.I.S.P.P. Archaeopress* (Oxford, 2009), pp. 145–151.

39. J. Trabska, A. Gawel, B. Trybalska, I. Fridrichová-Sýkorová, "Coloured raw materials on the Becov I site and in the vicinity. Preliminary results and futher perspectives," in *Ecce Homo Memoriam Jan Fridrich,* (Verlag Beir & Berna, 2010), pp. 205–217.

40. L. Dayet, J.-P. Faivre, F.-X. Le Bourdonnec, E. Discamps, A. Royer, E. Claud, C. Lahaye, N. Cantin, E. Tartar, A. Queffelec, B. Gravina, A. Turq, F. d'Errico, Manganese and iron oxide use at Combe-Grenal (Dordogne, France): A proxy for cultural change in Neanderthal communities. *J. Archaeol. Sci. Rep.* **25**, 239–256 (2019).

41. P. Bodu, H. Salomon, M. Leroyer, H.-G. Naton, J. Lacarriere, M. Dessoles, An open-air site from the recent Middle Palaeolithic in the Paris Basin (France): Les Bossats at Ormesson (Seine-et-Marne). *Quat. Int.* **331**, 39–59 (2014).

42. A. Pitarch Martí, F. d'Errico, Seeking black., Geochemical characterization by PIXE of Palaeolithic manganese-rich lumps and their potential sources. *J. Anthropol. Archaeol.* **50**, 54–68 (2018).

43. M. Soressi, W. Rendu, P.-J. Texier, É. Claud, F. d'Errico, V. Laroulandie, B. Maureille, M. Niclot, S. Schwortz, A.-M. Tillier, "Pech-de-l'Azé I (Dordogne, France): Nouveau regard sur un gisement moustérien de tradition acheuléenne connu depuis le XIXe siècle," in *Les sociétés Paléolithiques d'un grand Sud-Ouest: Nouveaux gisements, nouvelles méthodes, nouveaux résultats. Actes des journées décentralisées de la SPF des 24–25 novembre 2006. Mémoire XLVII de la Société préhistorique française. Société Préhistorique française* (2008), pp. 95–132. https://shs.hal.science/halshs-00432326. [Pech-de-l'Azé I (Dordogne, France): A new look at a Mousterian site of Acheulean tradition known since the 19th century].

44. M. Soressi, F. d'Errico, "Pigments, gravures, parures: les comportements symboliques controversés des Néandertaliens," in *Les Néandertaliens. Biologie et cultures* (Éditions du CTHS, CTHS, 2007), pp. 297–309. https://shs.hal.science/halshs-00444101. [Pigments, engravings, ornaments: The controversial symbolic behaviors of Neanderthals].

45. D. Peyrony, *Le Moustier: Ses gisements, ses industries, ses couches géologiques.* (Librairie Emile Nourry, 1930). [Le Moustier: Its deposits, its industries, its geological layers].

46. P.-Y. Demars, Les colorants dans le Moustérien du Périgord. L'apport des fouilles de F. Bordes. *Préhistoire Ariégeoise* **47**, 185–194 (1992). [Colorants in the Mousterian of the Périgord. The contribution of F. Bordes' excavations].

47. J. Zilhão, D. E. Angelucci, E. Badal-García, F. d'Errico, F. Daniel, L. Dayet, K. Douka, T. F. G. Higham, M. J. Martínez-Sánchez, R. Montes-Bernárdez, S. Murcia-Mascarós, C. Pérez-Sirvent, C. Roldán-García, M. Vanhaeren, V. Villaverde, R. Wood, J. Zapata, Symbolic use of marine shells and mineral pigments by Iberian Neandertals. *Proc. Natl. Acad. Sci. U.S.A.* **107**, 1023–1028 (2010).

48. N. Kraybill, "Pre agricultural tools for the preparation of foods in the Old World," in *Origins of Agriculture* (De Gruyter Mouton, 1977); https://doi.org/10.1515/9783110813487.485.

49. M. Peresani, M. Vanhaeren, E. Quaggiotto, A. Queffelec, F. d'Errico, An ochered fossil marine shell from the Mousterian of Fumane Cave, Italy. *PLOS ONE* **8**, e68572 (2013).

50. L. Dayet, F. d'Errico, R. Garcia-Moreno, Searching for consistencies in Châtelperronian pigment use. *J. Archaeol. Sci.* **44**, 180–193 (2014).

51. H. Salomon, Y. Coquinot, L. Beck, C. Vignaud, M. Lebon, G. P. Odin, F. Mathis, M. Julien, "Stratégies spécialisées d'acquisition de pigments rouges durant le Châtelperronien de la grotte du Renne à Arcy-sur-Cure (Yonne, France)," in *Paléo* (Amis du Musée National de Préhistoire et de la Recherche Archéologique, 2014), vol. Special issue. https://orbi.uliege.be/handle/2268/108354. [Specialized strategies for acquiring red pigments during the Châtelperronian of Grotte du Renne at Arcy-sur-Cure (Yonne, France)].

52. F. Caron, F. d'Errico, P. D. Moral, F. Santos, J. Zilhão, The reality of Neandertal symbolic behavior at the Grotte du Renne, Arcy-sur-Cure, France. *PLOS ONE* **6**, e21545 (2011).

53. M. Soressi, F. d'Errico, "Les Néandertaliens. Biologie et cultures," *CTHS* (CTHS, 2007), pp. 297–309. [The Neanderthals. Biology and cultures].

54. P. J. Heyes, K. Anastasakis, W. de Jong, A. van Hoesel, W. Roebroeks, M. Soressi, Selection and use of manganese dioxide by Neanderthals. *Sci. Rep.* **6**, 22159 (2016).

55. A. Pitarch Martí, F. d'Errico, J. Zilhão, Cueva de Ardales: Un caso de estudio para comprender el papel simbólico de las cuevas en el Paleolítico medio. *Takurunna* **10-11**,

219–241 (2021). [Cueva de Ardales: A case study for understanding the symbolic role of caves in the Middle Paleolithic].

56. E. M. Kolpakov, The late Acheulian site of Dashtadem-3 in Armenia. *PaleoAnthropology* **2009**, 3–31 (2009).

57. K. Paddayya, An Acheulian occupation site at Hunsgi, Peninsular India: A summary of the results of two seasons of excavation (1975–6). *World Archaeol.* **8**, 344–355 (1977).

58. M. Petraglia, R. Korisettar, N. Boivin, C. Clarkson, P. Ditchfield, S. Jones, J. Koshy, M. M. Lahr, C. Oppenheimer, D. Pyle, R. Roberts, J.-L. Schwenninger, L. Arnold, K. White, Middle Paleolithic assemblages from the Indian subcontinent before and after the Toba super-eruption. *Science* **317**, 114–116 (2007).

59. C. Clarkson, C. Harris, B. Li, C. M. Neudorf, R. G. Roberts, C. Lane, K. Norman, J. Pal, S. Jones, C. Shipton, J. Koshy, M. C. Gupta, D. P. Mishra, A. K. Dubey, N. Boivin, M. Petraglia, Human occupation of northern India spans the Toba super-eruption ~74,000 years ago. *Nat. Commun.* **11**, 961 (2020).

60. M. C. Langley, C. Clarkson, S. Ulm, Symbolic expression in Pleistocene Sahul, Sunda, and Wallacea. *Quat. Sci. Rev.* **221**, 105883 (2019).

61. E. E. Wreschner, "Red ochre, the transition between Lower and Middle Paleolithic and the origin of modern man," in *The Transition from Lower to Middle Palaeolithic and the Origins of Modern Man: International Symposium to Commemorate the 50th Anniversary of Excavations in the Mount Carmel Caves by D. A. E. Garrod*, A. Ronen, Ed. (BAR International Series, University of Haïfa, 1982), vol. 151, pp. 35–40.

62. N. E. Belousova, E. P. Rybin, A. Y. Fedorchenko, A. A. Anoykin, Kara-Bom: New investigations of a Palaeolithic site in the Gorny Altai, Russia. *Antiquity* **92**, e1 (2018).

63. A. P. Derevianko, A. V. Postnov, E. P. Rybin, Y. V. Kuzmin, S. G. Keates, The Pleistocene peopling of Siberia: A review of environmental and behavioural aspects. *Bull. Indo. Pac. Prehistory Assoc.* **25**, 57–68 (2005).

64. Ancient coloured ‘pencil’ up to 50,000 years old found in Siberia. https://www.ancient-origins.net/news-evolution-human-origins/denisova-pencil-0011176.

65. Z. Li, L. Doyon, H. Li, Q. Wang, Z. Zhang, Q. Zhao, F. d’Errico, Engraved bones from the archaic hominin site of Lingjing, Henan Province. *Antiquity* **93**, 886–900 (2019).

66. A. Pitarch Martí, Y. Wei, X. Gao, F. Chen, F. d’Errico, The earliest evidence of coloured ornaments in China: The ochred ostrich eggshell beads from Shuidonggou Locality 2. *J. Anthropol. Archaeol.* **48**, 102–113 (2017).

67. F.-G. Wang, S.-X. Yang, J.-Y. Ge, A. Ollé, K.-L. Zhao, J.-P. Yue, D. E. Rosso, K. Douka, Y. Guan, W.-Y. Li, H.-Y. Yang, L.-Q. Liu, F. Xie, Z.-T. Guo, R.-X. Zhu, C.-L. Deng, F. d’Errico, M. Petraglia, Innovative ochre processing and tool use in China 40,000 years ago. *Nature* **603**, 284–289 (2022).

68. Y. G. Kolosov, *Mousterian Sites of Belogorsk Region* (Kiev, Naükova Dümka, 1983).

69. Y. G. Kolosov, *Ak-Kajskaya musterskaya kultura* (Kiev, Naükova Dümka, 1986). [The Ak-Kaya Mousterian Culture (Russian: Ak-Kajskaya musterskaya kultura)].

70. V. N. Stepanchuk, *Lower and Middle Palaeolithic of Ukraine* (Cernovtsy, Zelena Bukovyna, 2006).

71. V. N. Stepanchuk, O. I. Nezdolii, D. O. Vietrov, Natural pigments in materials of the multilayered Mousterian site of Prolom II. *Archaeol. Early Hist. Ukr.* **28**, 7–21 (2018).

72. S. Ryzhov, V. N. Stepanchuk, O. Nezdolii, D. Vietrov, Analysis of micro-residues on stone tools from Zaskelna IX, Crimea: First results. *Arheologìa* 1, 5–25 (2022).

73. V. N. Stepanchuk, “Pebbles with ochre residues from Neanderthal sites of Eastern Crimea,” in *A Life Dedicated to the Paleolithic: Studies in Honorem Marin Cârciumaru* (Cetatea de Scaun, 2022), pp. 53–69.

74. L. Wadley, Ochre crayons or waste products? Replications compared with MSA ‘crayons’ from Sibudu Cave, South Africa. *Before Farming* **3**, 1-12 (2005).

75. C. S. Henshilwood, F. d'Errico, K. L. van Niekerk, Y. Coquinot, Z. Jacobs, S.-E. Lauritzen, M. Menu, R. Garcia-Moreno, A 100,000-year-old ochre-processing workshop at Blombos Cave, South Africa. *Science* **334**, 219–222 (2011).

76. A. Margalida, M. S. Braun, J. J. Negro, K. Schulze-Hagen, M. Wink, Cosmetic colouring by bearded vultures *Gypaetus barbatus*: Still no evidence for an antibacterial function. *PeerJ* **7**, e6783 (2019).

77. E. M. Pigott, T. Uthmeier, V. Chabai, T. Higham, The late Middle and Early Upper Paleolithic in Crimea (Ukraine) - A review of the Neanderthal refugium hypothesis, *J. Paleolit. Archaeol.* **7**, 27 (2024).

78. A. Majkić, F. d'Errico, V. Stepanchuck, Assessing the significance of Palaeolithic engraved cortexes. A case study from the Mousterian site of Kiik-Koba, Crimea. *PLOS ONE* **13**, e0195049 (2018).

79. A. Majkić, S. Evans, V. N. Stepanchuk, A. Tsvelykh, F. d'Errico, A decorated raven bone from the Zaskalnaya VI (Kolosovskaya) Neanderthal site, Crimea. *PLOS ONE* **12**, e0173435 (2017).

80. I. Watts, Red ochre, body painting, and language: Interpreting the Blombos ochre. *Cradle Lang.* **2**, 93–129 (2009).

81. S. Moyo, D. Mphuthi, E. Cukrowska, C. S. Henshilwood, K. Van Niekerk, L. Chimuka, Blombos Cave: Middle Stone Age ochre differentiation through FTIR, ICP OES, ED XRF and XRD. *Quat. Int.* **404**, 20–29 (2016).

82. R. F. Rifkin, Processing ochre in the Middle Stone Age: Testing the inference of prehistoric behaviours from actualistically derived experimental data. *J. Anthropol. Archaeol.* **31**, 174–195 (2012).

83. T. Hodgskiss, Identifying grinding, scoring and rubbing use-wear on experimental ochre pieces. *J. Archaeol. Sci.* **37**, 3344–3358 (2010).

84. A. Queffelec, F. d'Errico, M. Vanhaeren, Analyse des blocs de matière colorante de Praileaitz I (Deba, Gipuzkoa). *Munibe Monogr. Anthropol. Archaeol. Ser.* **1**, 493–503 (2017). [Analysis of coloring material blocks from Praileaitz I (Deba, Gipuzkoa)].

85. H. J. Lucas-Tooth, B. J. Price, A mathematical method for the investigation of interelement effects in x-ray fluorescence analysis. *Metallurgia* **64**, 149–152 (1961).

86. L. Dayet, F.-X. Le Bourdonnec, F. Daniel, G. Porraz, P.-J. Texier, Ochre provenance and procurement strategies during the Middle Stone Age at Diepkloof Rock Shelter, South Africa. *Archaeometry* **58**, 807–829 (2016).

87. A. M. Zipkin, S. H. Ambrose, J. M. Hanchar, P. M. Piccoli, A. S. Brooks, E. Y. Anthony, Elemental fingerprinting of Kenya Rift Valley ochre deposits for provenance studies of rock art and archaeological pigments. *Quat. Int.* **430**, 42–59 (2017).

88. L. Dayet, Invasive and non-invasive analyses of ochre and iron-based pigment raw materials: A methodological perspective. *Minerals* **11**, 210 (2021).

89. G. Mauran, B. Caron, L. Beck, F. Détroit, C. Noûs, O. Tombret, D. Pleurdeau, J.-J. Bahain, M. Lebon, Standardization procedure to provide a unified multi-method elemental compositional dataset, application to ferruginous colouring matters from Namibia. *J. Archaeol. Sci. Rep.* **43**, 103454 (2022).

90. R Core Team, R: A language and environment for statistical computing. R Foundation for Statistical Computing, (Vienna, Austria, 2022). http://www. R-project. org/.

91. H. Wickham, *Ggplot2: Elegant Graphics for Data Analysis.* (Springer, 2016); https://ggplot2. tidyverse.org.

92. S. Lê, J. Josse, F. Husson, FactoMineR: An R package for multivariate analysis. *J. Stat. Softw.* **25**, 1–18 (2008).

93. A. Kassambara, Ggpubr: "ggplot2" based publication ready plots. R Package Version 0.6.0 (2023); https://CRAN.R-project.org/package=ggpubr.

94. A. Kassambara, F. Mundt, Factoextra: Extract and visualize the results of multivariate data analyses. R Package Version 1.0.7 (2020), vol. 1. https://CRAN.R-project.org/package=factoextra.

95. C. O. Wilke, Cowplot: Streamlined plot theme and plot annotations for 'Ggplot2.' R Package Version 0.9.2 (2020); https://CRAN.R-project.org/package=cowplot.

96. V. N. Stepanchuck, "Studying the Lower and Middle Palaeolithic of Ukraine: Main trends, discussions and results," in *Prehistoric Ukraine: From the First Hunters to the First Farmers* (Oxbow Books, 2020), pp. 7–61.

97. Y. G. Kolosov, V. N. Stepanchuk, New type of Middle Palaeolithic industry in Eastern Crimea. *Arceologické Rozhl* **49**, 124–145 (1997).

98. V. Chabai, T. Uthmeier, New excavations at the Middle Paleolithic site Zaskalnaya V, Crimea. The 2012 and 2013 field seasons: A preliminary report. *Quartär* **64**, 27–71 (2017).

99. Y. G. Kolosov, "Ak-Kaya Mousterian sites and some results of their investigation," in *Issledovanie Paleolita v Krymu (1879–1979)* (Naükova Dümka, 1979), pp. 33–35.

100. Y. G. Kolosov, V. N. Stepanchuk, V. Chabai, *The Early Paleolithic of the Crimea.* (Naükova Dümka, 1993).

101. Y. G. Kolosov, V. N. Stepanchuk, New radiocarbon dates of Crimean Palaeolithic sites. *Kam'iana doba Ukrainy* **1**, 18–29 (2002).

102. E. I. Danilova, "The occipital bone of Neanderthal man from the Zaskalnaya V trench near Ak-Kaya," in *Palaeolithic Studies in Crimea.* (Naükova Dümka, 1979), pp. 76–84.

103. Z. P. Gubonina, "Environmental conditions of Palaeolithic man in the foothills of the Crimea," in *Palinologiya Chetvertichnogo Perioda.* (Nauka, 1985), pp. 95–103.

104. E. I. Danilova, Anthropological description of bone remains of Neanderthal children from the cultural layer III of the Mousterian site of Zaskalnaya VI (Crimea). *Vopr. Antropol.* **71**, 72–87 (1983).

105. Y. G. Kolosov, V. N. Stepanchuk, Crimean assemblages with bifacial tools: Brief review. *Etudes Rech. Archeol. Université de Liége* **95**, 265–274 (2000).

106. V. N. Gladilin, *Les problèmes du Paléolithique Inférieur de l'Europe orientale* (Naükova Dümka, 1976). [Problems of the Lower Paleolithic of Eastern Europe].

107. V. Chabai, A. E. Marks, M. Otte, Variability of Middle and Early Upper Palaeolithic of the Crimea. *Arheologìa* **4**, 19–47 (1998).

108. E. I. Danilova, "Neanderthal metacarpal fragment," in *Palaeolithic Studies in Crimea* (Kiev, Naükova Dümka, 1979), pp. 84–85.

109. V. N. Stepanchuk, N. N. Kovalyukh, J. Van der Plicht, Radiocarbon age of the Late Pleistocene Paleolithic sites of Crimea. *Stone Age of Ukraine* **5**, 34–61 (2004).

110. C. McKinney, J. Rink, "The absolute chronology of the Middle Paleolithic of the Crimea," paper presented at the 61st Annual Meeting of the Society of American Archaeology (New Orleans, April 11, 1996).

111. A. A. Velichko, "Geoecology of the Mousterian in East Europe and the adjacent areas," in *L'Homme de Neandertal*, M. Otte, Ed. (Liege, 1988), vol. 2, pp. 181–206.

112. A. A. Velichko, V. P. Dushevsky, P. D. Podgodetskiy, "The Zaskalnaya V and Zaskalnaya VI sites," in *Archaeology and Paleogeography of the Early Paleolithic of the Crimea and the Caucasus*, A. A. Velichko, N. D. Praslov, Eds. (Nauka, Moscow, 1978), pp. 20–37.

113. A. B. Klimchuk, E. I. Timokhina, G. N. Amelichev, V. Dublianskii Iu, K. Shpetl, *Hypogenic Karst of the Foothill Crimea and Its Geomorphologic Role* (Simferopol, DIAIPI, 2013).

114. V. N. Stepanchuck, Zh. M. Matviishina, S. Ryzhov, S. P. Karmazinenko, "Early man. Paleogeography and Archeology," (Kiev, Naükova Dümka, 2013).

115. Y. A. Smirnov, *Mousterian Burials in Eurasia* (Moscow, Nauka, 1991).

116. V. P. Yakimov, V. M. Kharitonov, "On the problem of the Crimean Neanderthals," in *Palaeolithic Studies in Crimea*, Y. G. Kolosov, Ed. (Kiev, Naükova Dümka, 1979), pp. 56–66.

117. Y. G. Kolossov, V. M. Kharitonov, V. P. Yakimov, "Palaeoanthropic specimens from the site Zaskalnaya VI in the Crimea," in *Paleoanthropology: Morphology and Paleoecology*, R. H. Tuttle, Ed. (De Gruyter Mouton, 1975), pp. 419–428.

118. V. N. Stepanchuck, Prolom II, a Middle Paleolithic cave site in the Eastern Crimea with non-utilitarian bone artefacts. *Proc. Prehist. Soc.* **59**, 17–37 (1993).

119. J. G. Enloe, F. David, G. Baryshnikov, Hyenas and hunters: Zooarchaeological investigations at Prolom II Cave, Crimea. *Int. J. Osteoarchaeol.*, 310–324 (2000).

120. Y. V. Kukharchuk, "Palaeolithic sites in the Romny region," in *Stone Age of Ukraine* (1996), vol. 1, pp. 30–42.

121. N. P. Herasymenko, O. S. Bonchkovskyi, Y. P. Rohozin, U. U, *Palaeoecology of Ancient Humans in the Territory of Ukraine (Palaeolithic)* (Kyiv, Print-Service, 2022).

122. A. E. E. Wreschner, R. Bolton, K. W. Butzer, H. Delporte, A. Häusler, A. Heinrich, A. Jacobson-Widding, T. Malinowski, C. Masset, S. Miller, A. Ronen, R. Solecki, P. H. Stephenson, L. L. Thomas, H. Zollinge, Red ochre and Human evolution: A case for discussion [and comments and reply]. *Curr. Anthropol.* **21**, 631–644 (1980).

123. A. E. E. Wreschner, "Evidence and interpretation of red ochre in the early prehistoric sequences," in *Hominid Evolution: Past, Present and Future,* A. R. Liss, (New York, 1985), pp. 387–394.

124. H. de Lumley, *Terra Amata, Nice, Alpes Maritimes, France* (CNRS Editions, 1969), vols. 1–2.

125. H. de Lumley, Les fouilles de Terra Amata à Nice. Premiers résultats. *Bull. Mus. d'Anthropologie Préhistorique Monaco* **13**, 29–51 (1966). [The excavations of Terra Amata in Nice. First results].

126. C. Peretto, M. Cremaschi, Les sols d'habitat du site paléolitique d'Isernia la Pineta (Molise, Italie Centrale). *Anthropologie* **92**, 1036 (1988). [Habitation floors of the Paleolithic site of Isernia la Pineta (Molise, Central Italy)].

127. K. Paddayya, Excavation of an Acheulian site at Hunsgi, South India. *Curr. Anthropol.* **17**, 760–761 (1976).

128. K. Paddayya, B. A. B. Blackwell, R. Jhaldiyal, M. D. Petraglia, S. Fevrier, D. A. Chaderton, J. I. B. Blickstein, A. R. Skinner, Recent findings on the Acheulian of the Hunsgi and Baichbal valleys, Karnataka, with special reference to the Isampur excavation and its dating. *Curr. Sci.* **83**, 641–647 (2002).

129. J. Ramos-Muñoz, P. Cantalejo, J. Blumenröther, V. Bolin, T. Otto, M. Rotgänger, M. Kehl, T. K. Nielsen, M. Espejo, D. Fernández-Sánchez, A. Moreno-Márquez, E. Vijande-Vila, L. Cabello, S. Becerra, Á. P. Martí, J. A. Riquelme, J. J. Cantillo-Duarte, S. Domínguez-Bella, P. Ramos-García, Y. Tafelmaier, G.-C. Weniger, The nature and chronology of human occupation at the Galerías Bajas, from Cueva de Ardales, Malaga, Spain. *PLOS ONE* **17**, e0266788 (2022).

130. M. Múzquiz Pérez-Seoane, "Análisis de los pigmentos de la Cueva del Castillo," in *El Origen Del Hombre Moderno En El Sur Oeste de Europa,* (Universidad Nacional de Educacion a Distancia, 1993), pp. 259–262. [Analysis of the pigments from Cueva del Castillo].

131. M. R. Sauter, *Les Industries Moustériennes et Aurignaciennes de La Station Paléolithique Du "Bonhomme" (Vallon Des Rebières, Dordogne)*, vol. 2 of *Cahier de préhistoire et d'archéologie* (Université de Genève, 1946). [The Mousterian and Aurignacian industries of the Paleolithic station of "Bonhomme" (Vallon des Rebières, Dordogne)].

132. C. San Juan, Les matières colorantes dans les collections du Musée National de Préhistoire des Eyzies. *PALEO Rev. Archéologie Préhistorique* **2**, 229–242 (1990). [Coloring materials in the collections of the National Museum of Prehistory at Les Eyzies].

133. D. de Sonneville-Bordes, Les industries moustériennes de l'abri Caminade-Est, commune de La Canéda (Dordogne). *Bull. Société Préhistorique Fr.* **66**, 18 (1969). [The Mousterian industries of the Caminade-Est rock shelter, commune of La Canéda (Dordogne)].

134. C. Couraud, Les pigments des grottes d'Arcy-sur-Cure (Yonne). *Gall. Préhistoire* **33**, 17–52 (1991). [Pigments from the caves of Arcy-sur-Cure (Yonne)].

135. D. Bonjean, Y. Vanbrabant, G. Abrams, S. Pirson, C. Burlet, K. Di Modica, M. Otte, J. Vander Auwera, M. Golitko, R. McMillan, E. Goemaere, A new Cambrian black pigment used during the late Middle Palaeolithic discovered at Scladina Cave (Andenne, Belgium). *J. Archaeol. Sci.* **55**, 253–265 (2015).

136. J. K. Kozlowski, M. Kaczanowska, *Studies on Raj Cave near Kielce (Poland) and Its Deposits* (Państwowe Wydawnictwo Naukowe, 1972), *Folia Quaternaria*.

137. M. Cârciumaru, M.-H. Moncel, M. Anghelinu, R. Cârciumaru, The Cioarei-Borosteni Cave (Carpathian Mountains, Romania): Middle Palaeolithic finds and technological analysis of the lithic assemblages. *Antiquity* **76**, 681–690 (2002).

138. M. Cârciumaru, E.-C. Niţu, O. Cîrstina, A geode painted with ochre by the Neanderthal man. *Comptes Rendus Palevol* **14**, 31–41 (2015).

139. L. Demay, S. Péan, M. Patou-Mathis, Mammoths used as food and building resources by Neanderthals: Zooarchaeological study applied to layer 4, Molodova I (Ukraine). *Quat. Int.* **276-277**, 212–226 (2012).

140. R. G. Klein, The Mousterian of European Russia. *Proc. Prehist. Soc.* **35**, 77–111 (1970).

141. L. Coutier, M. Emetaz, Station-des Rochettes (Dordogne). *Bull. Mém. Société Anthropol. Paris* **7**, 145–148 (1926).

142. V. N. Stepanchuck, I. Sapozhnikov, The Middle and Upper Pleistocene of Ukraine: A synopsis of Paleolithic finds with special reference to patterns of peopling and cultural development. *Arheol. Mold.* **33**, 13–24 (2010).

143. R. E. M. Hedges, R. A. Housley, P. B. Pettitt, C. B. Ramsey, G. J. V. Klinken, Radiocarbon dates from the Oxford AMS System: Archaeometry datalist 21. *Archaeometry* **38**, 181–207 (1996).
